# Telelactation Services and Breastfeeding by Race and Ethnicity: A Randomized Clinical Trial

**DOI:** 10.1001/jamanetworkopen.2024.61958

**Published:** 2025-02-27

**Authors:** Lori Uscher-Pines, Kandice Kapinos, Molly Waymouth, Khadesia Howell, Gaby Alvarado, Kristin Ray, Jill Demirci, Ateev Mehrotra, Rhianna Rogers, Kortney Floyd James, Maria DeYoreo

**Affiliations:** 1 RAND Corporation, Arlington, Virginia; 2 RAND Corporation, Santa Monica, California; 3 School of Medicine, University of Pittsburgh, Pittsburgh, Pennsylvania; 4 University of Pittsburgh School of Nursing, Pittsburgh, Pennsylvania; 5 School of Public Health, Brown University, Providence, Rhode Island

## Abstract

**Importance:**

There are substantial disparities in breastfeeding rates by race and ethnicity. Telelactation services that connect parents to lactation consultants via video are increasingly available and may reduce inequities in breastfeeding support; however, there is limited evidence on effectiveness.

**Objective:**

To assess the impact of video telelactation services on breastfeeding duration and exclusivity across a racially and ethnically diverse population of parents.

**Design, Setting, and Participants:**

This digital randomized clinical trial was conducted across 39 states. Eligible participants were aged 18 years or older, were in their third trimester of pregnancy with their first child, and intended to breastfeed. Participants were recruited through pregnancy apps from July 2021 to December 2022 and followed up through 24 weeks post partum. Analyses were completed from December 2023 to June 2024.

**Intervention:**

Pregnant individuals were randomized 1:1 to the treatment arm, which received access to a smartphone app with on-demand access to lactation consultants 24 hours a day via video visits, or to the control arm, which received an infant care e-book.

**Main Outcomes and Measures:**

Any breastfeeding and any infant formula use at 24 weeks post partum and time to breastfeeding cessation. The intention-to-treat analysis used regression models to estimate unadjusted effects and effects adjusted for baseline characteristics.

**Results:**

A total of 2108 participants were randomized, and 1911 were analyzed for primary outcomes. The mean (SD) age at the time of recruitment was 29.61 (5.37) years, and 707 participants (37.0%) had a household income of less than $55 000. In all, 611 participants (32.0%) identified as Black, 678 (35.5%) as Latinx, and 622 (32.5%) as races and/or ethnicities other than Black or Latinx. Among treatment arm participants, 466 (48.8%) used telelactation services. In intention-to-treat analyses, the proportion of all participants who reported any breastfeeding at 24 weeks was 674 (70.6%) in the treatment group and 639 (66.8%) in the control group (adjusted difference, 3.6 percentage points; 95% CI, −0.5 to 7.6 percentage points; *P* = .08), and the proportion with exclusive breastfeeding at 24 weeks was 447 (46.9%) in the treatment arm and 421 (44.1%) in the control arm (adjusted difference, 2.4 percentage points; 95% CI, −1.9 to 6.8 percentage points; *P* = .28). Differences were significant among Black participants. The proportion of Black participants who reported any breastfeeding at 24 weeks was 196 of 301 (65.1%) in the treatment arm and 178 of 310 (57.4%) in the control arm (adjusted difference, 7.5 percentage points; 95% CI, 0.2-14.8 percentage points; *P* = .045) and who reported breastfeeding exclusively at 24 weeks was 128 of 300 (42.7%) in the treatment arm and 105 of 310 (33.9%) in the control arm (adjusted difference, 9.2 percentage points; 95% CI, 1.4-16.9 percentage points; *P* = .02).

**Conclusions and Relevance:**

In this randomized clinical trial, telelactation services had no significant effect on breastfeeding rates among participants overall, but significant improvements were found among Black individuals. The results suggest that telelactation services could be a component of a comprehensive strategy to reduce racial disparities in breastfeeding rates.

**Trial Registration:**

ClinicalTrials.gov Identifier: NCT04856163

## Key Points

**Question:**

Does virtual breastfeeding support through telelactation services improve breastfeeding rates in racially and ethnically diverse populations?

**Findings:**

In this randomized clinical trial of 2108 birthing individuals, 71% who received telelactation services vs 67% of control individuals reported any breastfeeding at 24 weeks post partum, and 47% who received telelactation vs 44% of controls reported not using any formula at 24 weeks (differences were not statistically significant). Differences across arms for any breastfeeding and use of formula at 24 weeks were statistically significant among Black participants.

**Meaning:**

The findings suggest that offering telelactation services may significantly improve breastfeeding rates among Black parents.

## Teaser

This randomized clinical trial investigates whether breastfeeding support from telelactation services delivered virtually improves breastfeeding duration and exclusivity in a racially and ethnically diverse population.

## Introduction

Sustained breastfeeding offers health benefits for birthing people and their infants.^1,2,3,4^ However, the majority of birthing people stop breastfeeding before it is recommended^5^ and do not meet their breastfeeding goals.^6^ Furthermore, breastfeeding initiation and duration rates are lower among racial and ethnic minoritized individuals; for example, only 49% of Black infants receive any breastmilk at 6 months compared with 61% of non-Hispanic White infants.^5^ Breastfeeding disparities require urgent action because populations with lower breastfeeding rates are less likely to benefit from the myriad health and economic benefits breastfeeding confers.^6^

Many factors contribute to racial and ethnic disparities in breastfeeding rates, including differences in social and cultural support, language and literacy barriers, racism, and acculturation.^7,8,9^ Differential geographic or financial access to International Board Certified Lactation Consultants (IBCLCs) may also contribute to breastfeeding disparities.^10,11^ A key priority in “The Surgeon General’s Call to Action to Support Breastfeeding” is to increase access to IBCLCs.^6^

IBCLC services increase breastfeeding duration and exclusivity.^12,13,14,15^ Telelactation services, which allow remote IBCLCs to connect with breastfeeding parents via video, may increase access and convenience because new parents can avoid traveling with their infants. Telelactation can also connect IBCLCs with populations that have historically had inadequate access and can improve access to racially or culturally concordant support.

Telelactation services became widespread during the COVID-19 pandemic, with 34% of birthing people in the US reporting telehealth visits with IBCLCs in 2020 and 2021.^16^ Many payers and public health programs now offer telelactation services to augment or replace on-site IBCLCs. Despite the proliferation of these services, little is known about their effect on breastfeeding rates, how effectiveness may differ across populations, and their potential as a tool to mitigate breastfeeding disparities.^17,18^

The Telehealth for Mothers to Improve Lactation Confidence (Tele-MILC) digital randomized clinical trial evaluated the impact of a telelactation app on breastfeeding duration and exclusivity and assessed differences in impact across Black individuals, Latinx individuals, and non-Black and non-Latinx (predominantly White) individuals.^18^ We hypothesized that by increasing access to professional breastfeeding support, telelactation services would improve breastfeeding duration and exclusivity, with greater impacts among Black and Latinx individuals.

## Methods

The Tele-MILC trial was a digital, parallel-design randomized clinical trial that was powered to explore heterogeneity of treatment effects by race and ethnicity. Complete details on the study design, which was informed by the equity-centered model,^19,20^ were previously published.^21,22^ The study was approved by the RAND institutional review board and was registered with ClinicalTrials.gov (NCT04856163). Informed consent was documented via electronic signature. This study adhered to the Consolidated Standards of Reporting Trials (CONSORT) reporting guideline for clinical trials, and the trial protocol is included in Supplement 1.

### Setting and Participants

Participants were recruited from July 2021 to December 2022. The goal was to recruit equal numbers of Black participants, Latinx participants, and participants of other races and ethnicities. Participants self-reported their race and ethnicity via survey at the time of recruitment. Categories included American Indian or Alaska Native, Asian, Black or African American (alone or in combination with other races and ethnicities; hereafter, Black), Latinx (alone or in combination with other races except Black; hereafter, Latinx), Native Hawaiian or Pacific Islander, Middle Eastern or North African, White, multiracial and/or multiple ethnicities, and other race (any not otherwise specified). Groups other than Black and Latinx were combined into a non-Black and non-Latinx category.

Recruitment occurred via popular pregnancy apps (Ovia, BabyCenter, and What to Expect) that offer pregnancy tracking tools and educational content to millions of users in the US. The study opportunity was advertised to active users (through dedicated emails and personal newsfeeds) in the 39 states and territories with fewer than 5 IBCLCs per 1000 births.^23^ It is unknown how many individuals viewed study materials, because most emails from these apps are not opened.

To enroll, participants opened the study advertisement, completed a screening survey that determined eligibility, and completed an e-consent process. Pregnant individuals were eligible to participate if they were aged at least 18 years, were pregnant with their first child, were in their third trimester of pregnancy, intended to breastfeed, and spoke English and/or Spanish. Exclusion criteria included nonsingleton pregnancy, planned newborn separation at birth, having been advised by a health care practitioner not to breastfeed, and police custody or incarceration. Participants were randomized into 1 of 2 study arms—telelactation app (treatment) or e-book (control)—and followed up through 24 weeks post partum. As described in our published protocol, we followed best practices to detect fraudulent enrollment.^21^

### Treatment Arm

Participants in the treatment arm received access to Pacify Health’s Health Insurance Portability and Accountability Act–compliant smartphone app. The app provided unlimited, on-demand telelactation visits. IBCLCs were available via video 24 hours a day and provided lactation counseling in English or Spanish. Participants chose whether and how often to use telelactation services.

### Control Arm

Control arm participants received care as usual. Although they did not receive access to the study app, they could receive lactation support from other sources as part of their usual care. In addition, participants in this arm received an e-book on infant care (*Your Baby’s First Year*^24^). We monitored for contamination by assessing whether control arm participants enrolled with the telelactation app during the study period and found no evidence of contamination.

### Randomization

Participants were randomized in a 1:1 ratio using a secure, computer-generated schedule prepared by the study’s biostatistician (MD). The randomization scheme used block randomization stratified by race and ethnicity. In the screening survey, participants were asked to self-identify their race and ethnicity as presented in the US census. For primary outcomes, we applied a series of trumping rules that defined mutually exclusive categories. Participants who indicated that they identified as Black were categorized as Black regardless of whether they selected additional races and ethnicities, including Latinx. Furthermore, participants who were not Black and who identified as Latinx ethnicity were categorized as Latinx regardless of whether they selected additional (other than Black) races and ethnicities. We also conducted sensitivity analyses to explore the impact of different categorization methods on primary outcomes (eTables 2-4 in Supplement 2).

### Study Assessments

Participants completed online surveys in English or Spanish at the time of enrollment, at 4 weeks post partum, and at 24 weeks post partum. Participants received a $20 gift card for completing each of the 3 surveys and $20 for completing the step associated with their study arm (total of $80).

The baseline survey included questions on demographics, health and digital literacy, and breastfeeding intentions. Later assessments captured study outcomes, uptake of the intervention, birth experiences, and various sources and modalities of breastfeeding support (eAppendix in Supplement 2).^25,26^

While it was infeasible to blind participants to the treatment group after enrollment, participants were blinded to study hypotheses and to which arm was the treatment arm. For example, during the recruitment process, we explained that the study was testing electronic resources for pregnancy and parenting. Also, remote IBCLCs delivering the intervention did not know which parents were part of the study.

### Outcomes

The primary outcomes self-reported in the 24-week survey included 2 measures of breastfeeding duration and 1 measure of exclusivity. These were (1) any breastfeeding in the past 7 days (yes, no) (duration), (2) absence of infant formula in the preceding 24 hours (yes, no) (exclusivity), and (3) newborn’s or infant’s age (in months) when they ceased to receive the participant’s own milk (duration).

### Sample Size

We sought to recruit 1800 participants who would complete the final assessment at 24 weeks post partum. This sample size would provide 80% power to detect an overall Cohen *h* effect size that was small and clinically significant (*h* = 0.13), corresponding to a difference of 6.3% between arms under the assumption that breastfeeding rates 24 weeks post partum in the control arm would be consistent with 2015 Centers for Disease Control and Prevention estimates.^27^

A sample size of 1800 participants (600 in each race and ethnicity subgroup) would achieve statistical power of 80% to detect heterogenous treatment effects by race and ethnicity via an interaction test.^28^ These calculations assumed the intervention would help Black and Latinx participants but not others, with treatment effects leading to increases in breastfeeding rates of 15% for Black participants, 15% for Latinx participants, and 0% for non-Black and non-Latinx participants. We also note that the effect size that could be detected in subgroup analyses (with 300 per arm in each subgroup) was also small (Cohen *h* = 0.23).

### Statistical Analysis

As per the protocol, we conducted an intention-to-treat (ITT) analysis and a 2-staged probit instrumental variable approach to adjust for treatment nonuse (ie, lack of telelactation app use among those randomized to receive it). The ITT approach presented in the Results section includes all study participants who completed the 24-week survey according to their randomized assignment regardless of use. eTable 6 in Supplement 2 also includes the results of the ITT approach with all participants as randomized, including those who were lost to follow-up.

The instrumental variable approach estimated the effect of use of telelactation services in participants who behaved according to their assigned treatment arm (average treatment effect among those who adhered to treatment). Treatment assignment was the instrument for telelactation use in stage 1 (randomization to treatment group affects breastfeeding outcomes only through telelactation use).^29,30^ In stage 2, we regressed breastfeeding outcomes on the instrumented exposure (estimated telelactation use) from stage 1.

In our main instrumental variable analysis, we defined use of the treatment as reported use of video visits through the telelactation app. However, because video visits with IBCLCs were common during the COVID-19 pandemic as part of usual care, we conducted a sensitivity analysis that applied a broader definition of telelactation use to include any video visits with IBCLCs among both arms regardless of source (the eMethods in Supplement 2 gives details).

For both the ITT and instrumental variable overall and subgroup analyses, we estimated unadjusted effects and adjusted effects using regression models to adjust for chance imbalance of baseline characteristics^31^ and to increase power. Covariates included maternal educational level, maternal race and ethnicity, health insurance coverage, presence of a chronic condition, and weeks of gestation at delivery (the eMethods in Supplement 2 gives additional details). When assessing heterogeneity of treatment effects by race and ethnicity, we interacted the treatment dummy with maternal racial and ethnic group.

For binary outcomes, unadjusted effects were calculated as differences in proportions between treatment and control participants. We used logistic (probit for the instrumental variable) regressions to estimate adjusted effects, including the aforementioned covariates. We calculated postestimation probabilities for each group for ease of interpretation.^32^ Time to breastfeeding cessation was modeled using a discrete-time survival model,^33^ which can be estimated by creating an expanded person-period dataset and applying logistic regression including a series of time (month) dummies and study arm as well as covariates used for adjusted analyses.^34^ The model treats time as discrete by specifying a baseline hazard with 1 parameter for each possible event time in the data. The estimated regression coefficient for the treatment study arm dummy variable was interpreted as a hazard odds ratio^33,34^ and provided the odds of stopping breastfeeding for the treatment group compared with the odds of stopping breastfeeding for the control group.

The analytic sample was restricted to participants who completed the 24-week survey and reported they were still living with their infant. In this complete case analysis, there was minimal missing data on survey items (<2% overall for the 24-week survey). Two observations were excluded from models examining exclusive breastfeeding due to item nonresponse. In adjusted models, item nonresponse was addressed for weeks of gestation using the missing indicator method.^35^

In sensitivity analyses, we used multiple imputation by chain equation methods to address missing data due to attrition. These analyses included all participants who were randomized, including those who were lost to follow-up. Our imputation model included the outcomes and all covariates used in adjusted analyses as well as additional covariates (income, marital status, rurality, plans to return to work in the first year, and cesarean delivery).

The statistical analysis was completed from December 2023 to June 2024 and was performed using Stata, version 17.0 (StataCorp LLC) and R, version 4.2.2 (R Project for Statistical Computing). A 2-sided level of statistical significance was set at *P* ≤ .05.

## Results

**Figure 1.**
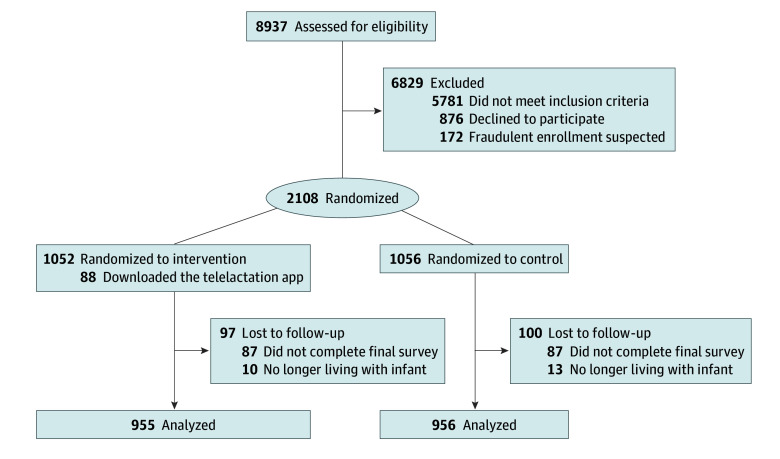
CONSORT Diagram

A total of 8937 individuals opened the study advertisement and were screened for eligibility, and 3137 were eligible for the trial (Figure 1). Of those, 1029 were not enrolled to maintain sample size targets for the 3 racial and ethnic groups. A total of 2108 participants were enrolled (1052 in the treatment arm and 1056 in the control arm); 1911 participants (956 in the control arm and 955 in the treatment arm) completed the final assessment, and 197 were lost to follow-up, resulting in an attrition rate of 9.3%. Among participants who completed the study, the mean (SD) age at the time of recruitment was 29.61 (5.37) years and 707 (37.0%) had a household income of less than $55 000. A total of 611 (32.0%) identified as Black, 678 (35.5%) identified as Latinx, and 622 (32.5%) identified as races and ethnicities other than Black or Latinx (58 [3.0%], American Indian or Alaska Native; 97 [5.1%], Asian; 19 [1.0%], Middle Eastern or North African; 15 [1.0%], Native Hawaiian or Pacific Islander; 983 [51.4%], White; 843 [44.1%], multiracial and/or multiple ethnicities; and 333 [17.4%], other race). Baseline characteristics were similar for control and intervention arm participants (Table 1). Details on how sample characteristics varied by race and ethnicity are included in eTable 1 in Supplement 2.

**Table 1.** Self-Reported Baseline Characteristics of the Analytic Sample

Characteristic^a^	Participants (N = 1911)^b^	
	Treatment arm (n = 955)	Control arm (n = 956)
Maternal characteristics		
Age, mean (SD), y	29.47 (5.22)	29.75 (5.52)
Married	589 (61.7)	553 (57.8)
Race and ethnicity (not mutually exclusive)		
American Indian or Alaska Native	35 (3.7)	23 (2.4)
Asian	49 (5.1)	48 (5.0)
Black or African American	301 (31.5)	310 (32.4)
Latinx	376 (61.7)	373 (57.8)
Middle Eastern or North African	12 (1.3)	7 (0.7)
Native Hawaiian or Pacific Islander	9 (0.9)	6 (0.6)
White	509 (53.3)	474 (49.6)
Multiracial and/or multiple ethnicities^c^	430 (45.0)	413 (43.2)
Other race^d^	157 (16.4)	176 (18.4)
Race and ethnicity (mutually exclusive with trumping rules applied)		
Black (including Black Latinx)	301 (31.5)	310 (32.4)
Latinx (non-Black)	342 (35.8)	336 (35.1)
Non-Black and non-Latinx^e^	312 (32.7)	310 (32.4)
Household income, $		
0-14 999	76 (8.0)	83 (8.7)
15 000-24 999	75 (7.9)	66 (6.9)
25 000-39 999	105 (11.0)	98 (10.3)
40 000-54 999	84 (8.8)	120 (12.6)
55 000-79 999	134 (14.0)	138 (14.4)
≥80 000	407 (42.6)	380 (39.7)
Unknown	74 (7.7)	71 (7.4)
Health insurance during pregnancy		
Medicaid or uninsured	303 (32.0)	316 (33.0)
Commercial or other	652 (68.0)	640 (67.0)
Completed educational level		
High school degree or less	124 (13.0)	122 (12.8)
Some college	269 (28.2)	287 (30.0)
4-y College	313 (32.8)	275 (28.8)
Graduate degree	249 (26.1)	272 (28.5)
Community type		
Large city	320 (33.5)	327 (34.2)
Suburb	340 (35.6)	343 (35.9)
Small city	245 (25.7)	238 (24.9)
Rural area	50 (5.2)	48 (5.0)
Lack of high-speed internet	20 (2.1)	31 (3.2)
Smoking during pregnancy	22 (2.4)	32 (3.5)
Prenatal comorbidities		
Mean (SD), No.	0.75 (1.04)	0.77 (1.08)
No. (%)	420 (44.0)	427 (44.7)
Expects to work in first year after birth		
Yes	673 (70.5)	684 (71.5)
No	123 (12.9)	119 (12.4)
Unsure or do not know	159 (16.6)	153 (16.0)
Infant and birth characteristics		
Infant sex		
Girl	436 (45.6)	455 (47.6)
Boy	505 (52.9)	489 (51.2)
Prefer not to disclose	14 (1.5)	12 (1.3)
Cesarean delivery	280 (30.4)	293 (32.1)
≥39 wk’ Gestation at delivery	692 (72.5)	685 (71.7)
Neonatal intensive care unit stay	96 (10.4)	104 (11.4)
Low birth weight (<2500 g)	45 (4.9)	38 (4.2)

^a^ The following measures had item nonresponse (sample sizes reflect the number of observations without missing values): smoking during pregnancy (n = 1835), Cesarean delivery (n = 1834), infant sex (n = 1811), and infant weight measures (n = 1834).

^b^ Data are presented as number (percentage) of participants unless otherwise indicated.

^c^ Self-identified as having 2 or more races and/or ethnicities.

^d^ Any race or ethnicity not otherwise specified.

^e^ Includes all racial and ethnic groups given under “Race and ethnicity (not mutually exclusive)” except for Black or African American and Latinx.

### Telelactation Use and Other Breastfeeding Support From IBCLCs

Among participants enrolled in the treatment group, 466 (48.8%) reported having video visits through the app: 156 of 301 Black participants (51.8%), 159 of 342 Latinx participants (46.5%), and 151 of 312 non-Black and non-Latinx participants (48.4%). Among these 466 app users, 186 (39.9%) reported participating in 1 video visit, 116 (24.9%) in 2 visits, and 164 (35.2%) in 3 or more visits. The number of visits did not vary significantly by race and ethnicity.

Use of (nonapp) video visits with external IBCLCs was not uncommon among control group participants. A total of 25 of 310 Black participants (8.1%), 37 of 336 Latinx participants (11.0%), and 28 of 311 non-Black and non-Latinx participants (9.0%) in the control group reported having video visits with external IBCLCs.

Among both treatment and control group participants, receipt of in-person IBCLC support in the 24 weeks after discharge varied by subgroup. A total of 218 Black participants (35.7%), 258 Latinx participants (38.1%), and 286 non-Black and non-Latinx participants (46.0%) reported receiving in-person IBCLC support (*P* = .004 by χ^2^ test). Participants in the treatment group were more likely than participants in the control group to receive any IBCLC care (including via telephone or video and in person) from hospital discharge through 24 weeks post partum: Black participants, 172 of 301 (57.1%) in the treatment arm vs 159 of 310 (51.3%) in the control arm (*P* = .14); Latinx participants, 215 of 342 (62.9%) in the treatment arm vs 162 of 336 (48.2%) in the control arm (*P* < .001); and non-Black and non-Latinx participants, 212 of 312 (68.0%) in the treatment arm vs 181 of 311 (58.2%) in the control arm (*P* = .01).

### ITT Analysis

The proportion of all participants (full sample) breastfeeding at 24 weeks was 674 (70.6%) in the treatment group and 639 (66.8%) in the control group (adjusted difference, 3.6 percentage points; 95% CI, −0.5 to 7.6 percentage points; *P* = .08) (Table 2). There was no evidence of heterogeneity in treatment effects for breastfeeding at 24 weeks, as the *P* value for the interaction was not statistically significant (*P* = .47).

**Table 2.** Treatment Effects for Any Breastfeeding and Exclusive Breastfeeding by Subpopulation in ITT and IV Analyses

Outcome	Participants, No./total No. (%)		Difference (95% CI), percentage points			
			ITT		IV	
	Telelactation arm	Control arm	Unadjusted	Adjusted^a^	Unadjusted	Adjusted^a^
**Any breastfeeding at 24 weeks**						
Black parents	196/301 (65.1)	178/310 (57.4)	7.7 (0.0 to 15.4)^b^	7.5 (0.2 to 14.8)^b^	15.6 (5.5 to 25.7)^c^	14.4 (4.9 to 23.9)^c^
Latinx parents	235/342 (68.7)	227/335 (67.8)	1.2 (−5.9 to 8.2)	1.1 (−5.8 to 8.1)	9.2 (−0.1 to 18.9)	7.9 (−1.7 to 17.5)
Non-Black and non-Latinx parents^d^	243/312 (77.9)	234/310 (75.5)	2.4 (−4.3 to 9.1)	2.3 (−4.7 to 9.3)	9.2 (0.3 to 19.1)^b^	8.5. (−0.7 to 17.7)
Full sample	674/955 (70.6)	639/956 (66.8)	3.7 (−0.4 to 7.9)	3.6 (−0.5 to 7.6)	11.1 (3.4 to 19.0)^c^	10.2 (2.6 to 17.8)^c^
**No formula use (exclusive breastfeeding) at 24 weeks**						
Black parents	128/300 (42.7)	105/310 (33.9)	8.8 (1.1 to 16.5)^b^	9.2 (1.4 to 16.9)^b^	15.0 (5.5 to 24.5)^c^	14.4 (4.9 to 23.9)^c^
Latinx parents	155/342 (45.3)	152/335 (45.4)	−0.1 (−7.6 to 7.5)	−0.4 (−7.8 to 7.0)	7.0 (−2.9 to 16.8)	5.5 (−4.2 to 15.3)
Non-Black and non-Latinx parents^d^	164/312 (52.6)	164/310 (52.9)	−0.3 (−8.2 to 7.5)	−0.9 (−8.6 to 6.8)	6.6 (−3.5 to 16.8)	5.0 (−5.0 to 15.0)
Full sample	447/955 (46.9)	421/956 (44.0)	2.8 (−1.7 to 7.2)	2.4 (−1.9 to 6.8)	9.3 (1.6 to 17.1)^b^	8.4 (0.5 to 15.7)^b^

Abbreviations: ITT, intent to treat; IV, instrumental variable.

^a^ Adjusted analyses included the following covariates: maternal educational level (4 categories), maternal race and ethnicity, an indicator for private health insurance during pregnancy, an indicator for whether the mother reported a chronic condition, and an indicator for delivery at 39 or more weeks’ gestation.

^b^ *P* < .05.

^c^ *P* < .01.

^d^ Includes American Indian or Alaska Native, Asian, Native Hawaiian or Pacific Islander, Middle Eastern or North African, White, multiracial and/or multiple ethnicities, and other race (any not otherwise specified).

The proportion of Black participants breastfeeding at 24 weeks was 196 of 301 (65.1%) in the treatment group and 178 of 310 (57.4%) in the control group (adjusted difference, 7.5 percentage points; 95% CI, 0.2-14.8 percentage points; *P* = .045). The proportion of Latinx participants breastfeeding at 24 weeks was 235 of 342 (68.7%) in the treatment group and 227 of 335 (67.8%) in the control group (adjusted difference, 1.1 percentage points; 95% CI, −5.8 to 8.1 percentage points; *P* = .75) (Table 2).

The proportion of all participants (full sample) who were breastfeeding exclusively at 24 weeks was 447 (46.9%) in the treatment group and 421 (44.1%) in the control group (adjusted difference, 2.4 percentage points; 95% CI, −1.9 to 6.8 percentage points; *P* = .28) (Table 2). There was no evidence of heterogeneity in treatment effects in exclusive breastfeeding at 24 weeks (*P* = .13 for interaction).

The proportion of Black participants who were breastfeeding exclusively at 24 weeks was 128 of 300 (42.7%) in the treatment group and 105 of 310 (33.9%) in the control group (adjusted difference, 9.2 percentage points; 95% CI, 1.4-16.9 percentage points; *P* = .02). The proportion of Latinx participants who were breastfeeding exclusively at 24 weeks was 155 of 342 (45.3%) and 152 of 335 (45.4%) in the treatment and control groups, respectively (adjusted difference, −0.4 percentage points; 95% CI, −7.8 to 7.0 percentage points; *P* = .92).

**Figure 2.**
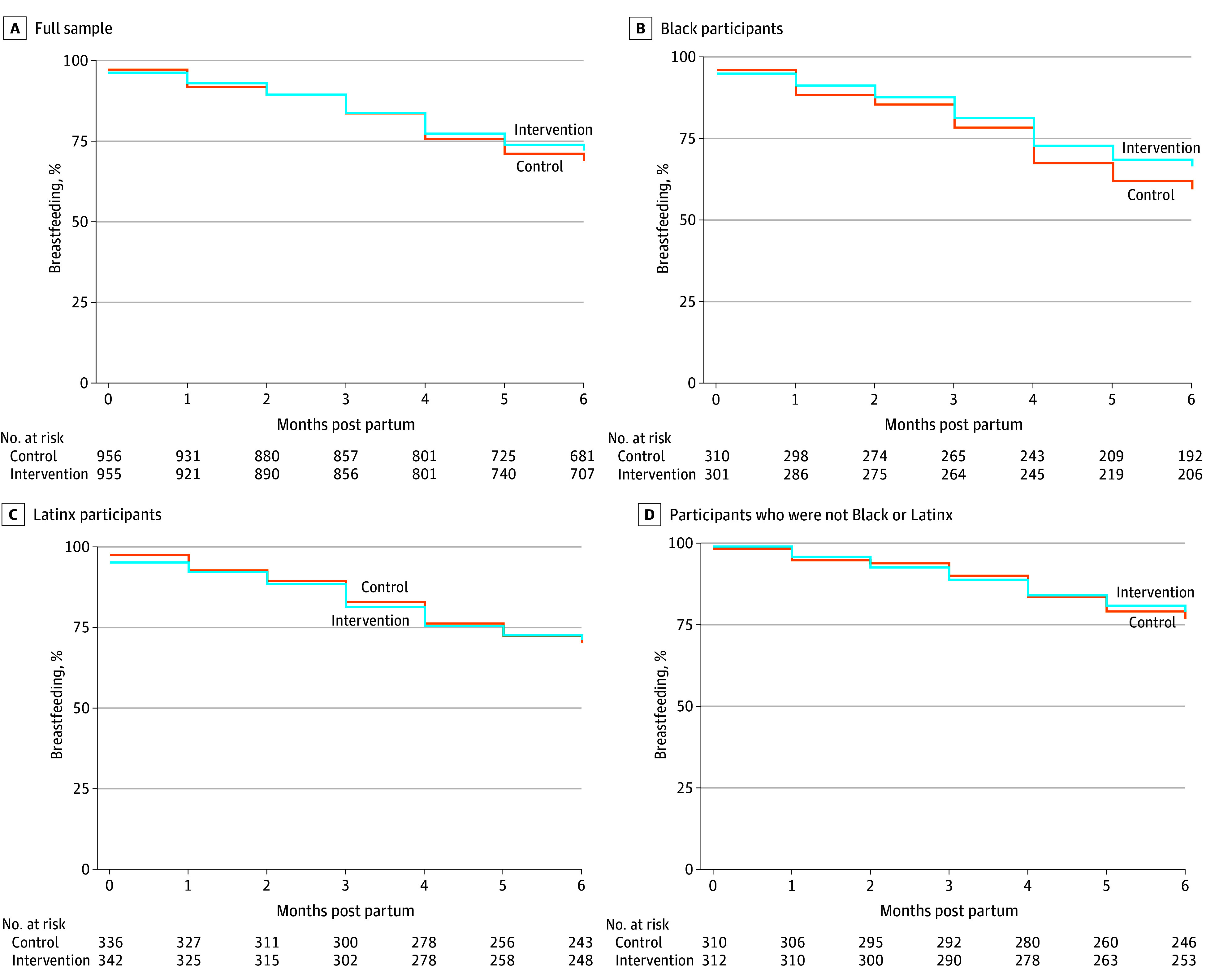
Proportion of Participants Breastfeeding by Month Following Birth

The estimated adjusted hazard odds ratios for the time to breastfeeding cessation in months were 0.89 (95% CI, 0.75-1.05) for all participants, 0.79 (95% CI, 0.60-1.04) for Black participants, 0.97 (95% CI, 0.73-1.29) for Latinx participants, and 0.94 (95% CI, 0.66-1.32) for non-Black and non-Latinx participants (Table 3 and Figure 2). Results for all outcomes based on multiple imputation of missing data rather than complete case analysis were similar; therefore, they are included in eTables 6 and 7 in Supplement 2.

**Table 3.** Treatment Effects for Time to Breastfeeding Cessation by Subpopulation in ITT and IV Analyses

Participants	Hazard odds ratio (95% CI)^a^	
	Unadjusted	Adjusted^b^
**ITT analysis**		
Black parents	0.79 (0.60-1.03)	0.79 (0.60-1.04)
Latinx parents	0.98 (0.74-1.31)	0.97 (0.73-1.29)
Non-Black and non-Latinx parents^c^	0.91 (0.64-1.28)	0.94 (0.66-1.32)
Full sample	0.88 (0.75-1.05)	0.89 (0.75-1.05)
**IV analysis**		
Black parents	0.63 (0.38-1.06)	0.67 (0.40-1.13)
Latinx parents	0.95 (0.52-1.74)	0.90 (0.49-1.64)
Non-Black and non-Latinx parents^c^	0.83 (0.41-1.66)	0.82 (0.41-1.60)
Full sample	0.77 (0.55-1.09)	0.78 (0.55-1.10)

Abbreviations: ITT, intent to treat; IV, instrumental variable.

^a^ Hazard odds ratios less than 1 indicate that the odds of stopping breastfeeding for treatment group participants were smaller than the odds of stopping breastfeeding for control group participants.

^b^ Adjusted analyses included the following covariates: maternal educational level (4 categories), maternal race and ethnicity, an indicator for private health insurance during pregnancy, an indicator for whether the mother reported a chronic condition, and an indicator for delivery at 39 or more weeks’ gestation.

^c^ Includes American Indian or Alaska Native, Asian, Native Hawaiian or Pacific Islander, Middle Eastern or North African, White, multiracial and/or multiple ethnicities, and other race (any not otherwise specified).

### Instrumental Variable Analysis

More than half of treatment arm participants did not use the telelactation app, which could dilute the true effect of the treatment. After adjusting for this nonuse, we found statistically significant improvements in both any breastfeeding and exclusive breastfeeding in the full sample of birthing individuals. Among all participants, the adjusted average treatment effect among those who adhered to treatment was 10.2 percentage points (95% CI, 2.6-17.8 percentage points; *P* = .008) for any breastfeeding and 8.4 percentage points (95% CI, 0.5-15.7 percentage points; *P* = .03) for exclusive breastfeeding. There was no evidence of heterogeneity of treatment effects for any breastfeeding (*P* = .43 for interaction) or exclusive breastfeeding (*P* = .15 for interaction) in adjusted instrumental variable models. In our sensitivity analyses using a broader measure of any telelactation use (study app video visits and external IBCLCs providing video visits) as the instrument, any breastfeeding and exclusive breastfeeding at 24 weeks increased by 18.4 percentage points (95% CI, 9.2-27.6 percentage points; *P* < .001) and 11.6 percentage points (95% CI, 1.9-21.4 percentage points; *P* = .02), respectively, among all participants in the treatment and control groups (eTable 5 in Supplement 2).

## Discussion

In this randomized clinical trial, there were no significant differences in breastfeeding duration or exclusivity in the full sample of individuals offered access to telelactation services in ITT analyses. However, it should be noted that the study was underpowered to detect differences of 2 to 4 percentage points. Rates of breastfeeding duration and exclusivity were higher among individuals who received access to the telelactation app, and when adjusting for use of telelactation services in prespecified instrumental variable analyses, we found statistically significant improvements in breastfeeding rates (8.4-10.2 percentage points) in the treatment arm vs the control arm. In prespecified subgroup analyses, telelactation app use led to larger increases among Black individuals, with significant and clinically meaningful improvements in any breastfeeding and exclusive breastfeeding at 24 weeks in both ITT and instrumental variable analyses.

We believe the instrumental variable analysis results are particularly relevant given challenges with nonuse of treatment in clinical trials of social and behavioral interventions. Although telelactation use in this study was robust compared with uptake of other digital health innovations in general population settings,^36^ thereby providing evidence of its acceptability, the rate of uptake we observed risks diluting potential treatment effects in an ITT analysis. The instrumental variable results suggest that telelactation services are positioned to have a meaningful impact on breastfeeding rates when paired with strategies to enhance use.

To our knowledge, this study is the first large trial of video telelactation services; however, other literature has shown that various digital interventions can be effective.^37,38^ Blackmore et al^38^ reviewed the impact of multiple virtual breastfeeding support interventions. Their systematic review concluded that virtual support may increase breastfeeding exclusivity at 6 months post partum and that the most successful virtual support interventions included frequent, sustained interactions with parents and personalization of content.^38^ The telelactation services provided to Tele-MILC participants included those features.

Our findings are also novel because we documented that telelactation services may have unique benefits for Black birthing people. We hypothesize that telelactation services may have larger benefits among Black individuals because they have lower baseline breastfeeding rates and may have reduced access to in-person support within usual care.^39^ As a result, offering telelactation services may address a gap in access to professional support that is perhaps more extreme among Black individuals. Our results demonstrate the importance of conducting trials with racially and ethnically diverse samples and that incorporate subgroup analyses.

To maximize the effectiveness of telelactation services as observed in the instrumental variable analysis, payers and public health programs should take steps to encourage use, including incorporating telelactation services into routine obstetric and pediatric care and promoting services in the context of trusted public health programs.^40,41^ To ensure equitable access, health systems and public health partners can ensure that telelactation platforms are secure and trustworthy, user friendly, and culturally responsive by codesigning services with racial and ethnic minoritized communities.^42^ Extra steps will need to be taken to ensure that populations impacted by the digital divide can access telelactation services. Promising practices include providing digital literacy training, increasing access to broadband and affordable cellular plans, and conducting demonstrations and dedicated support with enrollment (eg, through digital navigator programs).^43,44^

### Limitations

This study has several key limitations. First, digital trials and recruitment through pregnancy apps may not reach individuals without reliable access to technology and digital literacy.^45^ Second, not all treatment group participants used the provided telelactation services. Lack of adjustment for this nonuse in the ITT estimates biased these findings toward the null, which we addressed through the instrumental variable analyses. Third, because we only enrolled individuals who intended to breastfeed, we were not able to assess the impact of telelactation on breastfeeding initiation or how offering telelactation impacts the decisions of parents who are more ambivalent about breastfeeding. Furthermore, in part because we enrolled individuals who intended to breastfeed, breastfeeding rates in the control group were higher than in general population surveys.^5^ Finally, outcomes were self-reported, which can introduce response bias.

## Conclusions

In this randomized clinical trial, use of a telelactation app did not significantly improve breastfeeding rates overall but increased breastfeeding duration and exclusivity among all parents in the instrumental variable analysis and among Black parents in the ITT analysis. Our results suggest that offering telelactation could be a component of a comprehensive strategy to reduce racial and ethnic disparities in breastfeeding rates. Future research should test standalone and multicomponent interventions to address the varied barriers to breastfeeding that racial and ethnic minoritized parents experience. Furthermore, future research should explore the cost-effectiveness of different models of telelactation to inform decisions about implementation and payment.

## Notes

Supplement 1.
Trial Protocol

Supplement 2.
eMethods.
**eTable 1.** Participant Demographics by Race and Ethnicity and Study Arm **eTable 2.** Treatment Effects for Any Breastfeeding and Exclusive Breastfeeding by Non–Mutually Exclusive, Detailed Race and Ethnicity Categories—ITT Analysis **eTable 3.** Treatment Effects for Any Breastfeeding and Exclusive Breastfeeding by Non–Mutually Exclusive, Detailed Race and Ethnicity Categories—IV Analysis **eTable 4.** Treatment Effects for Time to Cessation of Breastfeeding by Non–Mutually Exclusive, Detailed Race and Ethnicity Categories—IV and ITT Analyses **eTable 5.** IV Analysis—Treatment Effects by Subgroup Using Broader Definition of Telelactation Use **eTable 6.** Complete Case Analysis vs Multiple Imputation Sensitivity Analysis for Any Breastfeeding and Exclusive Breastfeeding by Subgroup—ITT Analysis **eTable 7.** Multiple Imputation Sensitivity Analysis for Treatment Effects for Time to Breastfeeding Cessation by Subgroup—ITT Analysis **eAppendix.** Survey Instruments

Supplement 3.
Data Sharing Statement
