# Supplementary material for: Telelactation Services and Breastfeeding by Race and Ethnicity: A Randomized Clinical Trial
Source: JAMA Netw Open. 2025 Feb 27;8(2):e2461958. doi: 10.1001/jamanetworkopen.2024.61958 (PMC11868977; doi:10.1001/jamanetworkopen.2024.61958)
Supplement: Supplement 1. — Trial Protocol [file jamanetwopen-e2461958-s001.pdf]

Protocol and Statistical Analysis

Protocol Synopsis

Brief Summary

The aims of this siteless RCT are to 1) Assess the impact of DTC telelactation on breastfeeding duration and exclusivity; 2) Assess the effectiveness of telelactation across Latina, Black, and White women and rural and urban women 3) Explore whether breastfeeding self-efficacy is a mediator for any observed improvements in breastfeeding outcomes; 4) Explore how acceptability of and experiences with DTC telelactation vary across subpopulations to guide future improvement of these services. The primary outcomes include breastfeeding duration and exclusivity. Breastfeeding satisfaction is a secondary outcome.

Narrative Study Description

We propose to conduct a pragmatic, parallel design RCT to generate evidence on the impact of DTC telelactation on breastfeeding duration and exclusivity among minority mothers and explore subgroup differences in effectiveness. The study will be mixed methods, and use a sequential explanatory design in which qualitative interviews are used to explain and contextualize findings from the quantitative outcomes analysis. We will recruit 1800-2400 (depending on attrition) primiparous, pregnant women age ≥18 who intend to breastfeed and live in the 17 states most underserved by IBCLCs. Recruitment will occur via Ovia, a pregnancy tracker mobile phone application (app) used by one million pregnant women in the U.S. annually. Women will be randomized to: 1) on-demand telelactation video calls on personal devices or 2) usual care. Breastfeeding outcomes will be captured via surveys and interviews and compared across racial and ethnic groups. This study will track mothers for six months, generating 1) quantitative data on the impact of telelactation and differences in effectiveness across racial and ethnic minority groups; and 2) rich qualitative data on the experiences of different subgroups of mothers with the intervention, including barriers to use, satisfaction, and strengths and limitations of this delivery model.

We will randomize participants to receive telelactation services or usual care in a 1:1 ratio using block randomization stratified by race/ethnicity (White, Black, Latina, Other). We use a 1:1 ratio to maximize power. We will prepare a computerized randomization tool which draws from spreadsheets of random numbers. The tool will immediately display group assignment following consent.

We first estimated the number of study participants needed to achieve 80% power in the primary, intent-to-treat analysis, assuming a type 1 error rate of 5%. We applied national estimates of breastfeeding rates among diverse women who had initiated breastfeeding, and estimated that approximately 50-60% of control group participants would be breastfeeding at 24 weeks. To detect a clinically significant difference of 10-percentage points in any breastfeeding at 24 weeks postpartum across the study arms (telelactation vs. usual care), we would need 355-390 participants in each study arm (710-780 participants total) if using either a test of equal proportions or testing significance of the "treatment" variable in a logistic regression model. This effect size corresponds to a clinically significant effect size within the range of published effect sizes for IBCLC interventions.

Next, because we also plan to examine whether intervention effects vary across specific racial and ethnic groups (White or Other, Black, and Latina) and for rural vs. urban participants, we estimated the number of participants needed for this aim. For this analysis, we will fit a logistic regression model that includes the treatment dummy and a vector of race dummies, as well as their interactions. An interaction test directly assesses differences in treatment effects between complementary subgroups and involves one statistical test irrespective of the number of subgroups. Statisticians have argued that this is a more appropriate test for examining whether treatment effects differ by subgroup as compared to stratified analyses which analyze each subgroup separately and have increased risk of obtaining a false positive result. We assumed that breastfeeding rates at 24 weeks among control group participants are consistent with current CDC estimates (62% for White, 59% for Latina, and 53% for Black participants) and that we will secure equal numbers of participants in each combination of race/ethnicity and treatment. To achieve statistical power of 80% to detect heterogeneous treatment effects by race/ethnicity (e.g., effect size of 15% for Black, 15% for Latina, and 0% for White

participants; or effect size of 0% for Black, 0% for Latina, and 15% for White participants), we would require 1800 participants After inflating the sample size to account for 25% dropout, which is common for siteless trials, we would need to enroll approximately 2400 participants (800 in each race/ethnicity subgroup). This sample size provides 80% power to detect an overall effect size as small as 6.5% across arms. If our final sample is representative of Ovia users in terms of rural vs. urban distribution, then 2400 participants will also provide sufficient power for testing whether treatment effects differ by rural vs. urban location. These sample sizes will also give us sufficient statistical power (>80%) for our additional planned analyses, including detecting the treatment effect in the instrumental variable analysis (which has similar sample size requirements) and an additional survival analysis within Aim 1, which uses information on the week each participant stopped breastfeeding. Further, because the inclusion of covariates generally increases power, our estimates are conservative given the analysis plan.

To ultimately enroll up to 2400 participants over 27 months, we will need to enroll 89 women per month. As of September 2019, Ovia reported that in any given week, it had ~4500 unique users at 33 weeks gestation in the 17 states, and this population largely reflects the overall population of pregnant women in these states. As such, we estimate that over the 27-month period, there will be a pool of 486,000 women (4,500 x 108 weeks) in the phase of pregnancy we are targeting. If we restrict advertising to first-time mothers (40%), the pool is reduced to 194,000, including approximately 45,000 (23%) Latina and 31,000 (16%) Black women. If we estimate that 80% of women initiate breastfeeding and another 4% may be excluded due to other factors (e.g., lack of valid email address, age <17), we are left with 147,000 women (5,444 per month) who are likely to be eligible. Thus, meeting our recruitment targets will require enrolling 1.6% of the likely eligible population that will receive email and within-app ads.

Intervention Type: Behavioral (e.g., Psychotherapy, Lifestyle Counseling)

Intervention Name: Telelactation (Virtual breastfeeding support via video calls)

Description: Participants in this arm will receive unlimited, on demand telelactation visits through Pacify's mobile phone application which is publicly available for download on Apple and Android devices. After consent and randomization, women in this arm will receive materials (including written materials and an instructional video) on how to download and use the Pacify app, and they will receive a one-time coupon code that will be entered to unlock unlimited, free visits for six months past their due date. Pacify IBCLCs are available 24 hours a day and within minutes of a visit request, and can support visits in English or Spanish. To participate in a telelactation, participants will need a smartphone or tablet. Participants will also need access to Wi-Fi or a 4G cellular network to initiate a telelactation visit.

Intervention Model: Parallel

Masking: Yes

Masking Individual: Care Provider

Allocation: Randomized

Outcome Measures

Outcome #1 Type: Primary

Outcome #1 Name: Breastfeeding duration

Outcome #1 Time Frame: 6 months postpartum

Outcome #1 Description: Any breastfeeding (yes/no) as reported by the participant at six months post-delivery; Time to cessation of breastfeeding in months (as measured by reported age of infant when completely stopped receiving breastmilk)

Outcome #2 Type: Primary

Outcome #2 Name: Breastfeeding exclusivity

Outcome #2 Time Frame: 6 months postpartum

Outcome #2 Description: Only breastmilk (yes/no) in an infant's diet at 6 months of age as reported by the study participant

Outcome #3 Type: Secondary

Outcome #3 Name: Breastfeeding satisfaction

Outcome Time Frame: 1 month and 6 months post-partum

Outcome #3 Description: Level of satisfaction with the experience of breastfeeding

Statistical Analysis

To assess the impact of telelactation, we will estimate the intervention effect sizes for primary binary outcomes including (1) any breastfeeding and (2) exclusive breastfeeding at 24 weeks. Our key independent variable will be an indicator variable equal to one for mothers randomized to receive telelactation and zero otherwise, but we will also use a count measure of the number of telelactation visits to test whether there is a dose-response effect. We will also look at breastfeeding duration as a time-to-event variable with the event being defined as cessation of all breastfeeding.

We will use an ITT approach as the primary approach to estimate the effect of the treatment. We will calculate and compare means and associated confidence intervals of our outcome variables testing for statistical differences across the intervention and control group. Next, we will fit logistic regression models for each binary outcome measure with an indicator for study arm as the main independent variable, with the regression coefficient reflecting the treatment effect. We will also account for covariates that may be predictive of the outcomes or potential confounders in the regression. It is likely that some data needed for our analyses will be missing (e.g., incomplete covariates and some attrition). To avoid biasing the results or excluding data, we will impute missing values prior to analysis using multiple imputation methods. We will create multiple imputed complete datasets to account for the uncertainty in the missing values, and combine estimates from completed datasets using standard multiple imputation combining rules.

Time to cessation of breastfeeding is another outcome of interest, providing a different measure of breastfeeding duration than the binary indicator of breastfeeding as of 24 weeks. Time to cessation is measured in weeks, and can be modeled using survival analytic (time to event) methods. In the Cox proportional hazards (CPH) model, the measure of effect is the hazard rate, which here is the probability of cessation up until the point of measurement (e.g., 4 weeks, 24 weeks). The hazard ratio can be used to compare the study arms, giving the ratio of the total number of observed to expected cessations in the telelactation group relative to the usual care group. First, we will plot the survival functions for the two study arms. We will test for equality of the two survival functions using a logrank test (appropriate for censored data); the null hypothesis for this test is that the two samples have the same survival function. Second, we will estimate a semi-parametric CPH model including a binary indicator for study arm as well as other covariates. The CPH model leaves the baseline hazard unspecified, but allows for the inclusion of covariates. A negative coefficient for the indicator of treatment would indicate that the treatment arm has a lower risk for cessation of breastfeeding. We will use similar censored data methods to estimate the effect of treatment on the age at which infants were first fed formula. A truncated count distribution is appropriate for age measured in weeks.

While traditional guidelines for RCTs recommend the primary approach be ITT, ITT ignores contamination, measuring the effect of assignment to treatment, rather than the effect of receiving a treatment. We recognize that only some of the women offered telelactation will use it; as such, we will also address: "what is the benefit of receiving the treatment?" Contamination due to women being in the treatment group but not receiving treatment can decrease power yielding smaller observed effect sizes than would be obtained if all women in the treatment group utilized the treatment. Therefore, to maximize power and estimate the effect of receiving treatment, we will implement an innovative approach specifically designed to address the issue of noncompliance to treatment in an RCT, called a contamination adjusted intent-to-treat (CA ITT) analysis. We will use an instrumental variable model (IV), with treatment assignment as the "instrument." Treatment assignment is highly correlated with treatment received (the explanatory variable in this IV analysis), but has no independent effect on the outcomes (e.g., breastfeeding status), satisfying the requirements for using IV.

We will determine whether the intervention effects differ by race/ethnicity. We will use similar analytic approaches as described above controlling for key baseline covariates and main effects for treatment status, but also will include interaction terms between treatment status and race/ethnicity to determine the effects of the intervention for Black (projected to be 33% of final sample), Latina (projected to be 33% of the final sample), and White and Other women.
