# Supplementary material for: Telelactation Services and Breastfeeding by Race and Ethnicity: A Randomized Clinical Trial
Source: JAMA Netw Open. 2025 Feb 27;8(2):e2461958. doi: 10.1001/jamanetworkopen.2024.61958 (PMC11868977; doi:10.1001/jamanetworkopen.2024.61958)
Supplement: Supplement 2. — eMethods. eTable 1. Participant Demographics by Race and Ethnicity and Study Arm eTable 2. Treatment Effects for Any Breastfeeding and Exclusive Breastfeeding by Non–Mutually Exclusive, Detailed Race and Ethnicity Categories—ITT Analysis eTable 3. Treatment Effects for Any Breastfeeding and Exclusive Breastfeeding by Non–Mutually Exclusive, Detailed Race and Ethnicity Categories—IV Analysis eTable 4. Treatment Effects for Time to Cessation of Breastfeeding by Non–Mutually Exclusive, Detailed Race and Ethnicity Categories—IV and ITT Analyses eTable 5. IV Analysis—Treatment Effects by Subgroup Using Broader Definition of Telelactation Use eTable 6. Complete Case Analysis vs Multiple Imputation Sensitivity Analysis for Any Breastfeeding and Exclusive Breastfeeding by Subgroup—ITT Analysis eTable 7. Multiple Imputation Sensitivity Analysis for Treatment Effects for Time to Breastfeeding Cessation by Subgroup—ITT Analysis eAppendix. Survey Instruments [file jamanetwopen-e2461958-s002.pdf]

Supplementary Online Content

Uscher-Pines L, Kapinos K, Waymouth M, et al. Telelactation services and breastfeeding by race and ethnicity: a randomized clinical trial. *JAMA Netw Open*. 2025;8(2):e2461958. doi:10.1001/jamanetworkopen.2024.61958

eMethods.

eTable 1. Participant Demographics by Race and Ethnicity and Study Arm

eTable 2. Treatment Effects for Any Breastfeeding and Exclusive Breastfeeding by Non–Mutually Exclusive, Detailed Race and Ethnicity Categories—ITT Analysis

eTable 3. Treatment Effects for Any Breastfeeding and Exclusive Breastfeeding by Non–Mutually Exclusive, Detailed Race and Ethnicity Categories—IV Analysis

eTable 4. Treatment Effects for Time to Cessation of Breastfeeding by Non–Mutually Exclusive, Detailed Race and Ethnicity Categories—IV and ITT Analyses

eTable 5. IV Analysis—Treatment Effects by Subgroup Using Broader Definition of Telelactation Use

eTable 6. Complete Case Analysis vs Multiple Imputation Sensitivity Analysis for Any Breastfeeding and Exclusive Breastfeeding by Subgroup—ITT Analysis

eTable 7. Multiple Imputation Sensitivity Analysis for Treatment Effects for Time to Breastfeeding Cessation by Subgroup—ITT Analysis

eAppendix. Survey Instruments

This supplementary material has been provided by the authors to give readers additional information about their work.

© 2025 Uscher-Pines L et al. *JAMA Network Open*.

eMethods

Details on Defining "Use of Telelactation"

In our main instrumental variable (IV) specification, we defined use of telelactation (the treatment) as participating in one or more video calls through the telelactation app among treatment arm participants only. As a sensitivity analysis, we also created a broader measure of telelactation use based on reports of video IBCLC support among participants in both arms.

The main measure of use reported in the manuscript does not account for other video visits with IBCLCs outside of the study app, whereas the alternative measure considers any video visits with IBCLCs as "telelaction use." In addition, our alternative measure accounts for potential contamination from those in the control group engaging with IBCLCs through video visits as well. We present IV results using this second measure as an instrument below in eTable 5.

Details on Adjusted Models

In adjusted regression models, we included the following covariates: a categorical measure of maternal education (high school degree or less, some college, 4-year college degree, or graduate degree); maternal race and ethnicity; an indicator for whether the birthing individual was covered by private health insurance during pregnancy; an indicator for whether the birthing individual reported any chronic conditions (see list below); and an indicator for whether the infant was delivered at 39 weeks of gestation or later.

Chronic conditions included: high blood pressure/hypertension, Type 1 diabetes, Type 2 diabetes, obesity, hyperthyroidism, hypothyroidism, asthma, depression, anxiety, and any other chronic condition not listed (write in option).

© 2025 Uscher-Pines L et al. *JAMA Network Open*.

When assessing heterogeneity of treatment effects by race and ethnicity, we interacted the treatment dummy with maternal race and ethnic group.

In eTable 1, we show sample descriptive statistics for each of the three mutually exclusive, racial and ethnic groups.

© 2025 Uscher-Pines L et al. *JAMA Network Open*.

eTable 1: Participant Demographics by Race and Ethnicity and Study Arm

	Black				Latinx				Non-Black & Non-Latinx			
	Treatment n = 301		Control n = 310		Treatment n = 342		Control n = 336		Treatment n =312		Control n = 310	
	n	%	n	%	n	%	n	%	n	%	n	%
Age	28.95	(5.80)	29.16	(5.83)	29.08	(5.29)	29.19	(5.39)	30.4	(4.38)	30.9	(4.92)
Married	118	39.2%	118	38.1%	212	62.0%	180	53.6%	259	83.0%	255	82.3%
Race and Ethnicity (Not Mutually Exclusive)												
American Indian or Alaskan Native	15	5.0%	11	3.5%	17	5.0%	9	2.7%	3	1.0%	3	1.0%
Asian	11	3.7%	6	1.9%	11	3.2%	9	2.7%	27	8.7%	33	10.6%
Black or African American	301	100.0 %	310	100%	0	0.0%	0	0.0%	0	0.0%	0	0.0%
Native Hawaiian or Pacific Islander	4	1.3%	2	0.6%	1	0.3%	2	0.6%	4	1.3%	2	0.6%
Middle Eastern or North African	2	0.7%	1	0.3%	4	1.2%	2	0.6%	6	1.9%	4	1.3%
White	43	14.3%	32	10%	188	55.0%	163	48.5%	278	89.1%	279	90.0%
Other	5	1.7%	2	0.6%	148	43.3%	173	51.5%	4	1.3%	1	0.3%
Latinx	34	11.3%	37	11.9%	342	100%	336	100%	0	0%	0	0%
Multiple races and ethnicities	78	25.9%	67	21.6%	342	100%	336	100%	10	3.2%	10	3.2%
Type of Community												
Large City	115	38.2%	129	41.6%	135	44.9%	128	41.3%	70	22.4%	70	22.6%
Suburb	97	32.2%	96	31.0%	108	35.9%	113	36.5%	135	43.3%	134	43.2%
Small City	78	25.9%	71	22.9%	80	26.6%	84	27.1%	87	27.9%	83	26.8%
Rural Area	11	3.7%	14	4.5%	19	6.3%	11	3.5%	20	6.4%	23	7.4%
Income												
$0 to $14,999	42	14.0%	53	17.1%	29	8.5%	24	7.1%	5	1.6%	6	1.9%

© 2025 Uscher-Pines L et al. *JAMA Network Open*.

$15,000 to $24,999	40	13.3%	28	9.0%	29	8.5%	29	8.6%	6	1.9%	9	2.9%
$25,000 to $39,999	43	14.3%	37	11.9%	31	9.1%	46	13.7%	31	9.9%	15	4.8%
$40,000 to $54,999	30	10.0%	46	14.8%	34	9.9%	40	11.9%	20	6.4%	34	11.0%
$55,000 to $79,999	41	13.6%	48	15.5%	54	15.8%	49	14.6%	39	12.5%	41	13.2%
$80,000 or more	81	26.9%	69	22.3%	132	38.6%	121	36.0%	194	62.2%	190	61.3%
I don't know/unsure	24	8.0%	29	9.4%	33	9.6%	27	8.0%	17	5.4%	15	4.8%
Health Insurance												
Medicaid or Uninsured	148	47.4%	155	50.0%	114	36.5%	120	38.7%	41	13.1%	41	13.2%
All Else	164	52.6%	157	50.6%	198	63.5%	192	61.9%	271	86.9%	271	87.4%
Completed Education												
HS degree or less	56	18.6%	59	19.0%	55	16.1%	34	10.1%	13	4.2%	29	9.4%
Some College	112	37.2%	109	35.2%	97	28.4%	124	36.9%	60	19.2%	54	17.4%
4 year College	76	25.2%	81	26.1%	111	32.5%	86	25.6%	126	40.4%	108	34.8%
Graduate Degree	57	18.9%	61	19.7%	79	23.1%	92	27.4%	113	36.2%	119	38.4%
Limited Internet Access	10	3.3%	7	2.3%	5	1.5%	18	5.4%	5	1.6%	6	1.9%
Smoking in Pregnancy	13	4.4%	17	5.8%	3	0.9%	8	2.5%	6	2.0%	7	2.3%
Prenatal Health Conditions												
Hypertension/high blood pressure	20	6.6%	17	5.5%	6	1.8%	5	1.5%	4	1.3%	10	3.2%
Diabetes	5	1.7%	9	2.9%	5	1.5%	3	0.9%	2	0.6%	1	0.3%
Obesity	51	16.9%	55	17.7%	44	12.9%	43	12.8%	35	11.2%	34	11.0%
Thyroid Condition	10	3.3%	8	2.6%	20	5.8%	18	5.4%	24	7.7%	23	7.4%
Asthma	50	16.6%	44	14.2%	35	10.2%	34	10.1%	30	9.6%	25	8.1%
Depression or Anxiety	63	20.2%	76	24.5%	63	20.2%	75	24.2%	88	28.2%	76	24.5%
Other	14	4.7%	21	6.8%	14	4.1%	18	5.4%	31	9.9%	22	7.1%
Expects to Work During First Year												

© 2025 Uscher-Pines L et al. *JAMA Network Open*.

Yes	213	70.8%	226	72.9%	223	65.2%	220	65.5%	237	76.0%	238	76.8%
No	25	8.3%	36	11.6%	48	14.0%	43	12.8%	50	16.0%	40	12.9%
Unsure/Doesn't know	63	20.9%	48	15.5%	71	20.8%	73	21.7%	25	8.0%	32	10.3%
Infant & Birth Characteristics												
Sex = boy	150	51.4%	151	52.6%	171	53.4%	176	55.5%	184	62.2%	162	54.2%
Cesarean Delivery	97	33.1%	102	35.1%	96	29.6%	108	33.3%	87	28.7%	83	27.8%
Weeks of gestation = 39+	202	67%	210	68%	257	75%	246	73%	233	75%	229	74%
NICU Stay	26	8.9%	35	12.0%	39	12.0%	37	11.4%	31	10.2%	32	10.7%
Low Birth Weight (< 2500 g)	21	7.2%	20	6.9%	13	4.0%	11	3.4%	11	3.6%	7	2.3%

© 2025 Uscher-Pines L et al. *JAMA Network Open*.

In eTable 2, we show treatment effects for the intent-to-treat (ITT) analysis for each of the (non-mutually exclusive) racial and ethnic groups. This table presents two primary outcomes: any breastfeeding and exclusive breastfeeding.

eTable 2: Treatment Effects for Any Breastfeeding and Exclusive Breastfeeding by Non-Mutually Exclusive, Detailed Race and Ethnicity Categories: ITT Analysis

			Unadjusted	Adjusted
	Treatment N	Control N	Between group difference % (95% CI)	Between group difference % (95% CI)
Any breastfeeding at 24 weeks				
Black	301	310	7.7% (0.0, 15.4%)	7.6% (0.2, 15.11%)
Latinx	376	373	2.9% (-3.8, 9.6%)	2.2% (-4.5, 8.8%)
White	509	474	1.2% (-4.4, 6.9%)	1.1% (-4.5, 6.7%)
Asian	49	48	8.7% (-7.4, 24.7%)	8.7% (-6.2, 23.7%)
Middle Eastern or North African	12	7	26.2% (-16.1, 68.5%)	33.9% (-0.02, 67.9%)
American Indian or Alaskan Native	35	23	13.3% (-9.3, 35.9%)	10.9% (-12.2, 33.9%)
Native Hawaiian, Samoan, Chamorro, other Pacific Islander	9	6	0% (-48.7, 48.7%)	Not estimable
Other	157	176	1.5% (-8.3, 11.2%)	1.0% (-9.0, 11.0%)
Multiple races and ethnicities	430	413	2.3% (-4.1,8.6%)	1.8% (-4.5, 8.10%)
Exclusive breastfeeding at 24 weeks				
Black	301	310	8.8% (1.1, 16.48%)	9.0% (1.5, 16.5%)
Latinx	376	373	0.6% (-6.5, 7.7%)	-0.1% (-7.1, 7.0%)
White	509	474	-0.8% (-7.0, 5.5%)	-1.0% (-7.1, 5.2%)
Asian	49	48	13.5% (-6.2, 33.1%)	14.5% (-3.9, 33.0%)
Middle Eastern or North African	12	7	46.4% (5.0, 87.9%)	45.1% (8.9, 81.3%)
American Indian or Alaskan Native	35	23	-0.6% (-26.7, 25.44%)	-2.2% (-29.2, 24.8%)
Native Hawaiian, Samoan, Chamorro, other Pacific Islander	9	6	27.8% (-16.3, 71.9%)	Not estimable
Other	157	176	0.1% (-10.6, 10.8%)	0.4% (-10.4, 11.2%)

© 2025 Uscher-Pines L et al. *JAMA Network Open*.

Multiple races and ethnicities	430	413	0.5% (-6.3, 7.2%)	-0.01% (-6.7, 6.7%)

**Multiple races/ethnicities is defined as self-identifying as having two or more races and/or ethnicities. In some models, the subgroup sample sizes were too small to be able to estimate regression models; we labeled those as “not estimable.”*

© 2025 Uscher-Pines L et al. *JAMA Network Open*.

In eTable 3, we show treatment effects for the instrumental variable analysis for each of the (non-mutually exclusive) racial and ethnic groups. This table presents two primary outcomes: any breastfeeding and exclusive breastfeeding.

eTable 3: Treatment Effects for Any Breastfeeding and Exclusive Breastfeeding by Non-Mutually Exclusive, Detailed Race and Ethnicity Categories: IV Analysis

	No (%)		Unadjusted	Adjusted
	Treatment	Control	Between group difference % (95% CI)	Between group difference % (95% CI)
Any breastfeeding at 24 weeks				
Black	301	310	13.3% (0.3, 26.3%)	12.7% (0.1, 25.3%)
Latinx	376	373	11.3 (-1.0, 23.4%)	9.7% (2.5, 21.8%)
White	509	474	8.3% (-2.1, 18.7%)	6.4% (-3.9, 16.6%)
Asian	49	48	33.2% (3.7, 62.7%)	26.2% (-0.1, 52.5%)
Middle Eastern or North African	12	7	Not estimable	Not estimable
American Indian or Alaskan Native	35	23	14.5% (-25.4, 54.3%)	9.8% (-32.4, 52.0%)
Native Hawaiian, Samoan, Chamorro, other Pacific Islander	9	6	Not estimable	Not estimable
Other	157	176	12.7% (-6.1, 31.4%)	13.0% (-6.6, 32.5%)
Multiple races/ethnicities	430	413	12.0% (0.3, 23.7%)	10.3% (-1.4, 22.1%)
Exclusive breastfeeding at 24 weeks				
Black	301	310	15.7% (2.7, 28.6%)	15.0% (2.4, 27.6%)
Latinx	376	373	10.8% (-2.2, 23.7%)	9.3% (-3.6, 22.1%)
White	509	474	0.8% (-10.7, 12.3%)	0.8% (-12.2, 10.5%)
Asian	49	48	49.4% (12.5, 86.3%)	40.9% (8.1, 73.7%)
Middle Eastern or North African	12	7	Not estimable	Not estimable
American Indian or Alaskan Native	35	23	Not estimable	Not estimable

© 2025 Uscher-Pines L et al. *JAMA Network Open*.

Native Hawaiian, Samoan, Chamorro, other Pacific Islander	9	6	Not estimable	Not estimable
Other	157	176	17.8% (-2.9, 38.5%)	20.8% (-0.6, 42.2%)
Multiple races/ethnicities	430	413	12.0% (-0.5, 24.5%)	10.0% (-2.4, 22.4%)

** Multiple races/ethnicities is defined as self-identifying as having two or more races and/or ethnicities. In some models, the subgroup sample sizes were too small to be able to estimate regression models; we labeled those as "not estimable."*

© 2025 Uscher-Pines L et al. *JAMA Network Open*.

In eTable 4, we show treatment effects for the ITT and IV analyses for each of the (non-mutually exclusive) racial and ethnic groups.

This table presents hazard odds ratios for the outcome of time to breastfeeding cessation.

eTable 4: Treatment Effects for Time to Cessation of Breastfeeding by Non-Mutually Exclusive, Detailed Race and Ethnicity Categories: IV and ITT Analyses

	Unadjusted Hazard Odds Ratio (95% CI)	Adjusted Hazard Odds Ratio (95% CI)
**Intent to Treat (ITT) Approach**		
Black	0.79 (0.60, 1.03)	0.78 (0.59, 1.23)
Latinx	0.90 (0.69, 1.19)	0.91 (0.69, 1.20)
White	0.98 (0.76, 1.25)	1.00 (0.78, 1.29)
Asian	0.64 (0.26, 1.60)	0.63 (0.24, 1.60)
Middle Eastern or North African	0.51 (0.06, 3.98)	0.43 (0.02, 11.40)
American Indian or Alaskan Native	0.64 (0.20, 2.04)	0.85 (0.23, 3.16)
Native Hawaiian, Samoan, Chamorro, other Pacific Islander	0.84 (0.13, 5.57)	1.40 (0.03, 64.19)
Multiple races and ethnicities	0.95 (0.73, 1.22)	0.95 (0.74, 1.24)
**Instrumental Variable (IV) Approach**		
Black	0.63 (0.38, 1.06)	0.62 (0.37, 1.04)

© 2025 Uscher-Pines L et al. *JAMA Network Open*.

Latinx	0.81 (0.46, 1.42)	0.87 (0.49, 1.54)
White	0.95 (0.56, 1.59)	0.89 (0.52, 1.52)
Asian	0.41 (0.07, 2.28)	0.15 (0.02, 1.36)
Middle Eastern or North African	Not estimable	Not estimable
American Indian or Alaskan Native	0.41 (0.05, 3.69)	0.63 (0.07, 5.55)
Native Hawaiian, Samoan, Chamorro, other Pacific Islander	Not estimable	Not estimable
Multiple races and ethnicities	0.88 (0.51, 1.52)	0.93 (0.54, 1.62)

**Multiple races and ethnicities is defined as self-identifying as having two or more races and/or ethnicities. In some models, the subgroup sample sizes were too small to be able to estimate regression models; we labeled those as "not estimable."*

© 2025 Uscher-Pines L et al. *JAMA Network Open*.

In eTable 5, we present results of the IV analysis for all primary outcomes that accounted for non-use among treatment arm participants and contamination among control arm participants using a broader definition of telelactation use. In this analysis, treatment arm participants were considered users of telelactation if they reported participating in any video visits with lactation consultants within or outside the study app. Also, control arm participants are considered users of telelactation (evidence of contamination) if they reported participating in any video visits with lactation consultants within or outside the study app. In adjusted models, we found no evidence of heterogenous treatment effects for any breastfeeding at 24 weeks (p=0.41) or exclusive breastfeeding at 24 weeks (p=0.17).

eTable 5: IV Analysis: Treatment Effects by Subgroup Using Broader Definition of Telelactation Use

Outcomes	No (%)		Unadjusted		Adjusted	
	Telelactation	Control	Between group difference % (95% CI)	Hazard Ratio	Between group difference % (95% CI)	Hazard Ratio
Any breastfeeding at 24 weeks						
Black	196 (65.1%)	178 (57.4%)	24.6% (13.6, 35.6%)	NA	21.8% (10.9, 32.7%)	NA
Latinx	235 (68.7%)	227 (67.6%)	18.6% (7.5, 29.7%)	NA	16.3% (5.2, 27.4)%	NA
Non-Black & Non-Latinx	243 (77.9%)	234 (75.5%)	17.0% (7.0, 27%)	NA	15.8% (5.4, 26.2)	NA
Full sample	674 (70.6%)	639 (66.8%)	20.3% (10.9, 29.7%)*	NA	18.4% (9.2, 27.6%)*	NA

© 2025 Uscher-Pines L et al. *JAMA Network Open*.

Exclusive breastfeeding at 24 weeks						
Black	128 (42.7%)	105 (33.9%)	18.7% (7.8, 29.6%)	NA	16.8% (5.7, 27.9%)	NA
Latinx	155 (45.3%)	152 (45.4%)	11.3% (-0.4, 23.0%)	NA	8.3% (-3.4, 20.1%)	NA
Non-Black & Non-Latinx	164 (52.6%)	164 (52.9%)	10.7% (-1.1, 22.5%)	NA	7.6% (-4.3, 19.4%)	NA
Full sample	447 (46.9%)	421 (44.1%)	14.1% (4.4, 23.8%)*	NA	11.6% (1.9, 21.4%)*	NA
Time to event of breastfeeding cessation						
Black	NA	NA	NA	0.61 (0.34, 1.06)	NA	0.62 (0.35, 1.09)
Latinx	NA	NA	NA	0.94 (0.48, 1.8)	NA	0.91 (0.46, 1.78)
Non-Black& Non-Latinx	NA	NA	NA	0.81 (0.35, 1.84)	NA	0.82 (0.36, 1.88)
Full sample	NA	NA	NA	0.75 (0.51, 1.10)	NA	0.75 (0.50, 1.11)

**Differences are statistically significant (p<.05). Statistical significance is only assessed for analyses on the full sample.*
*** Hazard odds ratios less than 1 indicate that treatment group participants were less likely than control group participants to stop breastfeeding.*

© 2025 Uscher-Pines L et al. *JAMA Network Open*.

In eTable 6, we show the results of the multiple imputation sensitivity analysis that addresses missing outcome data among the 9% of trial participants who did not complete the study. This table compares odds ratios for the complete case analysis (analysis presented in the manuscript) and the sensitivity MICE analysis. It presents ITT analyses for two primary outcomes: any breastfeeding and exclusive breastfeeding.

eTable 6: Complete Case Analysis vs. Multiple Imputation Sensitivity Analysis for Any Breastfeeding and Exclusive Breastfeeding by Subgroup, ITT Analysis

	Completed Case Analysis		MICE Analysis (5 samples)			
	**Unadjusted ORs (95% CI)**	**Adjusted± ORs (95% CI)**	Treatment N	Control N	**Unadjusted ORs (95% CI)**	**Adjusted± ORs (95% CI)**
Any Breastfeeding at 24 weeks						
Black	1.21 (0.74, 1.99)	1.24 (0.75, 2.05)	343	350	1.14 (0.68, 1.91)	1.16 (0.69, 1.97)
Latinx	0.92 (0.56, 1.51)	0.93 (0.56, 1.54)	385	381	0.92 (0.56, 1.50)	0.93 (0.56, 1.54)
Non-Black and Non-Latinx	ref	ref	324	325	ref	ref
Full Sample	1.19 (0.98, 1.45)	1.19 (0.98, 1.45)	1052	1056	1.15 (0.94, 1.40)	1.16 (0.94, 1.42)
No Formula Use (Exclusive breastfeeding) at 24 weeks						
Black	1.47 (0.93, 2.32)	1.54 (0.97, 2.44)	343	350	1.38 (0.89, 2.15)	1.43 (0.91, 2.25)
Latinx	1.01 (0.65, 1.57)	1.02 (0.65, 1.59)	385	381	1.04 (0.67, 1.60)	1.05 (0.68, 1.63)
Non-Black and Non-Latinx	ref	ref	324	325	ref	ref
Full Sample	1.12 (0.93, 1.34)	1.11 (0.92, 1.33)	1052	1056	1.10 (0.92, 1.32)	1.10 (0.92, 1.32)

±*Adjusted analyses included the following covariates: maternal education (5 categories), maternal race and ethnicity, an indicator for private health insurance during pregnancy, an indicator for whether the mother reported a chronic condition, and an indicator for delivery at 39 or greater weeks of gestation. Subgroup analyses also included interaction terms between maternal race/ethnicity and treatment dummy.*

© 2025 Uscher-Pines L et al. *JAMA Network Open*.

In eTable 7, we show the results of the multiple imputation sensitivity analysis that addresses missing outcome data among the 9% of trial participants who did not complete the study. This table presents results of the MICE analysis. It presents ITT analyses for the time to breastfeeding cessation outcome.

eTable 7: Multiple Imputation Sensitivity Analysis for Treatment Effects for Time to Breastfeeding Cessation by Subgroup, ITT Analysis

	Unadjusted Hazard Odds Ratio (95% CI)	Adjusted Hazard Odds Ratio (95% CI)
ITT Analysis		
Black	0.83 (0.63 1.10)	0.83 (0.63 1.10)
Latinx	0.96 (0.71, 1.30)	0.95 (0.70, 1.29)
Non-Black& Non-Latinx	0.90 (0.64, 1.27)	0.91 (0.64, 1.28)
Full sample	0.89 (0.76, 1.06)	0.89 (0.75, 1.06)

±*Adjusted analyses included the following covariates: maternal education (5 categories), maternal race and ethnicity, an indicator for private health insurance during pregnancy, an indicator for whether the mother reported a chronic condition, and an indicator for delivery at 39 or greater weeks of gestation. Subgroup analyses also included interaction terms between maternal race/ethnicity and treatment dummy.*

© 2025 Uscher-Pines L et al. *JAMA Network Open*.

eAppendix: Survey Instruments

The eligibility survey, baseline survey, 4-week survey, and 24-week survey instruments that participants completed are included below.

Eligibility Survey

Please complete this 5-minute survey to see if you are eligible to participate in the study. After you complete all questions, you will be told if you are eligible. Eligible individuals will then go through a brief consent process where we will explain how we will protect your privacy throughout the study. After you learn more, you can decide if you'd like to join the study.

If you are NOT eligible to participate based on your responses, please do not try to take the survey again. The study team is checking to make sure that individuals only try to sign up once.

Let's Find Out if you are Eligible…

1) How many weeks pregnant are you now? (Please round down when you answer. For example, if you are 34 weeks and 6 days pregnant, check 34 weeks.)

    A. 32 weeks pregnant or less [Programming note-ineligible]
    B. 33 weeks pregnant
    C. 34 weeks pregnant
    D. 35 weeks pregnant
    E. 36 weeks pregnant
    F. 37 weeks pregnant
    G. 38 or more weeks pregnant [Programming note-ineligible]
    H. Don't know/not sure [Programming note-ineligible]

2) What is your baby's due date? [Programming Note- Nothing is ineligible here; all dates accepted. However, there will be a flag calculated for dashboard. Flag if parent who reports 33 weeks has a due date that is not between 40-60 days out; if parent who reports 34 weeks has a due date that is not between 30-50 days out; if a parent who reports 35 weeks has a due date that is not between 25-45 days out]

    __ ___ ____
    Day Month Year

3) Are you pregnant with more than one baby (e.g., twins or triplets)?
    A. Yes [Programming note: ineligible]
    B. No

4) How many other babies have you had? Do not include the baby you are expecting.
    A. None

© 2025 Uscher-Pines L et al. *JAMA Network Open*.

B. 1 [Programming note: ineligible]
C. 2 or more [Programming note: ineligible]

5) How do you plan to feed your new baby in the first month after birth?
   A. Breast milk only (my baby will not be given formula)
   B. Formula only (none of my own milk) [Programming note: ineligible]
   C. Both breast milk and formula
   D. I don't know yet/not sure [Programming note: ineligible]

6) Please rate how much you agree with the following statement:

When my baby is born, I am planning to give breastfeeding a try. Would you say you….

   A. Very much agree
   B. Somewhat agree
   C. Unsure [Programming note: ineligible]
   D. Somewhat disagree [Programming note: ineligible]
   E. Very much disagree [Programming note: ineligible]

7) Has a healthcare professional told you that you should <u>not</u> breastfeed your new baby for a medical reason? For example, some mothers who are undergoing chemotherapy, use drugs or take certain medications, or are HIV positive are advised not to breastfeed.
   A. Yes [Programming note: ineligible]
   B. No

8) Do you think you will be separated from your baby for more than one week during their first month of life for any reason? For example, some mothers have to deploy with the military or plan to place their baby up for adoption.
   A. Yes [Programming note: ineligible]
   B. No

9) At this time, are you in police custody or incarcerated?

   A. Yes [Programming note: ineligible]
   B. No

10) How old are you? [Programming note- This should be drop down. Ineligible if not between 18-45 but drop down should include 15-65]

   ____

© 2025 Uscher-Pines L et al. *JAMA Network Open*.

11) What state do you currently live in? [Programming note: Must be Alabama, Arkansas, DC, Florida, Georgia, Kentucky, Louisiana, Michigan, Mississippi, Nevada, North Dakota, South Dakota, Tennessee, Texas, Utah, West Virginia, or Wyoming. If not U.S., exclude; drop down should include non-U.S. option.]

(State drop down)______

Please answer BOTH Question 12 about Hispanic origin and Question 13 about race. For this survey, Hispanic origins are not races.

12) Are you of Hispanic, Latino, or Spanish origin? Check ALL that apply.

A. No, not of Hispanic, Latino, or Spanish origin
B. Yes, Mexican, Mexican American, Chicano
C. Yes, Puerto Rican
D. Yes, Cuban
E. Yes, another Hispanic, Latino, or Spanish origin (for example, Salvadoran, Dominican, Colombian, Guatemalan, Ecuadorian)

13) What is your race? Check ALL that apply.

A. Black or African American (for example, African American, Nigerian, Haitian)
B. Middle Eastern or North African (for example, Ethiopian, Lebanese, Egyptian)
C. American Indian or Alaskan Native (for example, Navajo Tribe, Blackfeet Tribe)
D. White (for example, German, Irish, English, Italian)
E. Asian (Chinese, Filipino, Vietnamese, Korean, Japanese, Asian Indian)
F. Native Hawaiian, Samoan, Chamorro, other Pacific Islander
G. Some other race

[If any ineligible answers]

Thank you for your interest. We are trying to get a mix of pregnant people from different places and with different experiences to participate in our study. Based on your responses, you are not eligible. Please do not try to take the eligibility survey again. Our study team is checking to make sure that individuals only complete the survey once.

[If all eligible answers]

Based on your responses, you are eligible to participate in the study. You will now be taken through an informed consent process where you learn more about the study. If you decide to participate after learning more, you will join the study and take your first survey.

© 2025 Uscher-Pines L et al. *JAMA Network Open*.

Baseline Survey

Introduction

This is the first of three surveys that you will complete over the next eight months as part of the Tele-MILC study. This survey asks about your background and plans for feeding your baby over the next few months. It also asks about your use of and experience with technology and with healthcare. After you complete the survey, you will receive a $20 gift card.

Please answer all questions honestly. There is no wrong way to answer these questions, and no answer will disqualify you from receiving your gift codes. If you have questions about your rights as a participant in this survey, or are not satisfied with any aspect of the survey, you may contact the study team at TeleMILC@rand.org.

1. What is your date of birth? [Programming note: Create flag for dashboard if age in screener does not match date of birth here]

   __ ___ ____
   Day Month Year

The next few questions ask about your thoughts about feeding your new baby.

2. Which of the following is closest to your opinion? The best way for me to feed my baby is to give him/her……

   A. Breast milk only (breastfeeding and/or pumped breast milk)
   B. Formula only
   C. A mix of both breast milk and formula
   D. No opinion

3. How old do you think your baby will be when you start feeding him or her formula on a regular basis (e.g., once a day or more)?

   A. Less than one month
   B. 1 to 2 months
   C. 3 to 5 months
   D. 6 to 9 months
   E. 10 or more months
   F. I never plan to feed my baby formula on regular basis
   G. I don’t know/not sure

© 2025 Uscher-Pines L et al. *JAMA Network Open*.

4. Consider the other people who may help care for your baby (for example, your partner, your mother, your mother-in-law). How do they think your baby should be fed in the first month after birth?

A. Only fed breast milk
B. Only fed formula
C. Fed both breast milk and formula
D. They don't have an opinion
E. I don't know their opinions
E. They don't agree/have different opinions about what my baby should be fed

Please rate how much you agree with the statements below about how you think breastfeeding will go after your baby is born.

5**.** I believe I will be able to meet my breastfeeding goals.

A. Strongly agree
B. Agree
C. Neither agree nor disagree
D. Disagree
E. Strongly disagree

6. I believe I will be satisfied with my breastfeeding experience.

A. Strongly agree
B. Agree
C. Neither agree nor disagree
D. Disagree
E. Strongly disagree

The next question asks about your health before you were pregnant.

7. **Before you were pregnant**, did a doctor or other health care worker tell you that you had any of the following health conditions? Check ALL that apply.

A. High blood pressure/hypertension
B. Type I diabetes
C. Type II diabetes
D. Obesity (BMI $\geq$ 30)
E. Hyperthyroidism (over-active thyroid)
F. Hypothyroidism (under-active thyroid)
G. Asthma
H. Depression
I. Anxiety

© 2025 Uscher-Pines L et al. *JAMA Network Open*.

J. Other chronic health condition, please specify-----[Programming note: Allow 250 characters]
K. None of these [Programming note: If this is selected, do not allow other selections]

The next question asks about your pregnancy so far.

8. **During your current pregnancy,** what kind of health insurance do you have to pay for your prenatal care? Check ALL that apply.

A. Private health insurance (for example, through an employer or union or through your state's health exchange)
B. Medicaid (health insurance program run by your state that provides coverage for lower-income people)
C. Medicare (federal health insurance program for people who are 65 or older and for certain younger people with disabilities)
D. Military health care (TRICARE/VA/CHAMPVA)
E. Indian Health Service
F. I have not had any health insurance coverage during my current pregnancy. [Programming note: If this is selected, do not allow other selections]

The next few questions ask about your height and weight.

9. What was your weight just before you became pregnant? Please give your best guess.

________pounds

10. How tall are you without shoes? Please give your best guess.

_____feet _____ inches

Because this study sends you surveys and messages through email and texts and provides online resources, the next few questions ask about your use of and comfort with technology.

11. Does your household currently subscribe to Internet service at home?

A. Yes
B. No

12. (If 11=A) What kind of Internet service do you have at home? Check ALL that apply.

A. Broadband (high speed) Internet service such as DSL, cable, or fiber optic service
B. Dial-Up Internet service
C. Satellite Internet service
D. Wireless Internet
E. Cellular data plan for a mobile phone or other device

© 2025 Uscher-Pines L et al. *JAMA Network Open*.

F. I don't know/not sure

The next few questions ask about how often you do certain activities on your mobile phone.

14. How often do you use video chatting programs (for example, Zoom, FaceTime)?
    A. Never
    B. Rarely
    C. Sometimes
    D. Often
    E. Always

15. How often do you use money transfer or payment apps (for example, Venmo, Apple Pay)?
    A. Never
    B. Rarely
    C. Sometimes
    D. Often
    E. Always

16. What do you like most about the Ovia pregnancy app? [Programming note: Allow 400 characters]

Please rate how much you agree with the statements below about technology companies. Examples of technology companies include Google, Facebook, and Amazon.

17. I can trust major technology companies to do what is right.

    F. Strongly agree
    G. Agree
    H. Neither agree nor disagree
    I. Disagree
    J. Strongly disagree

18. I think technology companies do enough to protect the personal data of their users.

    A. Strongly agree
    B. Agree
    C. Neither agree nor disagree
    D. Disagree
    E. Strongly disagree

19. Overall, technology companies have had a negative impact on me personally.

    A. Strongly agree
    B. Agree
    C. Neither agree nor disagree

© 2025 Uscher-Pines L et al. *JAMA Network Open*.

D. Disagree
E. Strongly disagree

The next few questions ask some basic things about you.

20. What is the highest degree or level of school you COMPLETED?

A. Grade school
B. Some high school
C. High school graduate or GED
D. Some college, no degree (1-3 years)
E. Associate degree in college (2 years)
F. Bachelor's degree (e.g. BA, AB, BS)
G. Graduate degree

21. What is your current marital status? Please check one.
A. Married
B. Living with partner, not married
C. Living apart from partner, not married
D. Single
E. Widowed
F. Divorced
G. Separated

22. How many people are currently living in your household? Please include all adults, children, and babies as well as yourself.

________________________[Programming note: Allow 1-25]

23. How often do you need to have someone help you when you read instructions, pamphlets, or other written material from your doctor or pharmacy?
A. Never
B. Rarely
C. Sometimes
D. Often
E. Always

24. Which of the following best describes the community you live in now?
A. A large city
B. A suburb near a large city
C. A small city or town
D. A rural area

© 2025 Uscher-Pines L et al. *JAMA Network Open*.

25. Do you speak a language other than English at home?

   A. Yes
   B. No

25. Which languages do you speak fluently? Check ALL that apply.

   A. English
   B. Spanish
   C. Mandarin
   D. French
   E. Arabic
   F. Other language

26. Do you plan to work for pay during your baby's first year?

   A. Yes
   B. No
   C. I don't know

27. (If 26=A) How old do you think your baby will be when you start working for pay? (This includes returning to an old job after you deliver or starting a new one, either full or part-time.)

   A. Less than 1 month old
   B. 1 to 2 months old
   C. 3 to 4 months old
   D. 5 to 6 months old
   E. More than 7 months old
   F. I don't know/not sure

28. **Over the last 12 months**, what was your total yearly household income before taxes? Include your income, your partner's income, and any other income you may have received such as alimony, child support, and financial assistance from the state or federal government. All information will be kept private. Would you say it was between…

   A. $0 to $14,999
   B. $15,000 to $24,999
   C. $25,000 to $$39,999
   D. $40,000 to $54,999
   E. $55,000 to $79,999
   F. $80,000 or more
   G. I don't know/not sure

Thank you for completing the survey! The research team will now review your responses. The research team reviews responses to make sure that real people complete the survey,

© 2025 Uscher-Pines L et al. *JAMA Network Open*.

and no one tries to sign up more than once. Once we have welcomed you to the study, we will send you a gift card in the form of a gift code. Expect your gift code to arrive via email within one week.

© 2025 Uscher-Pines L et al. *JAMA Network Open*.

Week 4 Survey

Introduction

This is the second of three surveys that you will be taking over the next five months as part of the Tele-MILC study. This survey asks about your experience feeding your baby over the four weeks since your baby's due date. After you complete the survey, you will receive a $20 gift card.

Please answer all questions honestly. There is no wrong way to answer these questions, and no answer will disqualify you from receiving your gift card.

If you have questions about your rights as a participant in this survey, or are not satisfied with any aspect of the survey, you may contact the study team at TeleMILC@rand.org.

1. Is your baby living with you now? (If you have been separated from your baby permanently or for many weeks due to work-related travel, adoption, death, or any other reason please mark "No.")

   A. Yes
   B. No

[IF Q1=B] This is the last question. Thank you for taking the time to complete this survey. Your $20 gift code will be sent to you shortly.

2. What is your baby's date of birth? [Programming note: Day Month Year]

__ ___ ____
Day Month Year

3. Is your baby a boy or a girl?

   A. Boy
   B. Girl
   C. Prefer not to say

4. How many weeks pregnant were you when your baby was born? (Please round down when you answer. For example, if you were 37 weeks and 6 days pregnant, check 37 weeks.)

   A. 33 weeks
   B. 34 weeks
   C. 35 weeks
   D. 36 weeks
   E. 37 weeks
   F. 38 weeks
   G. 39 weeks
   H. 40 weeks
   I. 41 or more (overdue)

© 2025 Uscher-Pines L et al. *JAMA Network Open*.

5. How was your baby delivered?

A. Vaginally
B. C-section (cesarean section)

6. Did your baby have to stay in an intensive care unit (NICU) at any time after birth?

A. Yes
B. No

7. What was your baby's weight at birth? Please give your best guess.

____pounds _______ounces

The next question asks about participation in WIC. WIC is a government program that gives food to pregnant people, parents, babies, and young children.

8. **In the past four months,** did you or your baby get any WIC benefits such as food, vouchers, or services?

A. Yes
B. No

The next few questions ask about feeding your new baby.

9. Was your baby ever breastfed or fed your breastmilk with a bottle, even for a short period of time?

A. Yes
B. No

10. (If 9=B) What were your reasons for <u>not</u> breastfeeding your baby? Check ALL that apply.

A. I was sick or on medicine
B. My baby was sick or had a medical issue
C. I had too many other duties or responsibilities
D. I couldn't get breastfeeding to work (e.g., baby wouldn't latch)
E. I didn't want to
F. I planned to go back to work or school
G. Other, please explain:------- [Programming note: Allow 300 characters]

11. (If 9=A) Since your baby was born, has he/she been fed any formula?

A. Yes
B. No

© 2025 Uscher-Pines L et al. *JAMA Network Open*.

12. (If 11=A) How old was your baby when he/she was first fed formula? Please give your best guess.

A. 1 day old or less
B. 2-7 days old
C. 8-14 days old
D. 15 or more days old

13. Which of the following has your baby been fed in the past week? Check ALL that apply.

A. My breast milk
B. Formula
C. Another person's breast milk (e.g., donor breast milk)

----

14. (If 13=A) How are you currently providing your breast milk to your baby?

A. Only feeding from the breast
B. Only giving pumped/expressed breast milk
C. A combination of feeding from the breast and giving pumped/expressed breast milk

15. (If 13=B) In the last 24 hours, what portion of your baby's diet was formula? Please give your best guess.

A. All (100% formula)
B. Almost all (75-99% formula or 1-7ounces of breast milk)
C. Most (50-74% formula or 8-12 ounces of breast milk)
D. Some (25-49% formula or 13-22 ounces of breast milk)
E. A little (less than 25% formula or 23 ounces or more of breast milk)
F. None (100% breast milk)

----

16. (If 13=B ONLY or 13=C ONLY or 13= B&C ONLY) How old was your baby when you completely stopped feeding him/her your breast milk? Please give your best guess.

___weeks old

17. (If 13=B ONLY or 13=C ONLY or 13=B&C ONLY) What were the main reason(s) you stopped breastfeeding? Check up to three reasons. [programming note: they can check less than three but three is the maximum]

A. My baby had difficulty sucking or latching
B. I thought my baby was not gaining enough weight
C. Breastfeeding was painful
D. Breastfeeding was too hard or time consuming
E. Breast milk alone did not satisfy my baby
F. I was not making enough milk or my milk dried up

© 2025 Uscher-Pines L et al. *JAMA Network Open*.

G. I had too many other duties or responsibilities
H. I felt it was the right time to stop breastfeeding
I. I got sick or I had to stop for medical reasons
J. I needed to change my diet
K. I needed to take a medication that interfered with breastfeeding
L. I started smoking or drinking alcohol
M. I had to go back to work or school
N. My baby got sick or had a medical issue
O. Family and/or friends told me I should stop breastfeeding
P. A doctor or other medical professional told me I should stop breastfeeding
Q. Other, please specify________ [Programming note: Allow 300 characters]

The next question asks about smoking.

18. **In the last month**, did you smoke, vape, or use e-cigarettes? Check ALL that apply.
   A. Yes, I smoked cigarettes
   B. Yes, I used a vape pen or used e-cigarettes (for example, Juul)
   C. No [Programming note: If this is selected, do not allow other selections]

(If 13=A) The next few questions ask about your level of confidence with breastfeeding your baby. Read each statement and check the answer that describes your level of confidence

19. (If 13=A) I can determine that my baby is getting enough breast milk.

A. Not at all confident
B. Not confident
C. Somewhat confident
D. Confident
E. Always confident

20. (If 13=A) I can successfully cope with breastfeeding like I have with other challenging tasks.

A. Not at all confident
B. Not confident
C. Somewhat confident
D. Confident
E. Always confident

21. (If 13=A) I can breastfeed my baby without using formula as a supplement.

A. Not at all confident
B. Not confident
C. Somewhat confident

D. Confident
E. Always confident

22. (If 13=A) I can ensure that my baby is properly latched on while breastfeeding.

A. Not at all confident
B. Not confident
C. Somewhat confident
D. Confident
E. Always confident

23. (If 13=A) I can manage breastfeeding to my satisfaction.

A. Not at all confident
B. Not confident
C. Somewhat confident
D. Confident
E. Always confident

24. (If 13=A) I can manage to breastfeed even if my baby is crying.

A. Not at all confident
B. Not confident
C. Somewhat confident
D. Confident
E. Always confident

25. (If 13=A) I continue to want to breastfeed.

A. Not at all confident
B. Not confident
C. Somewhat confident
D. Confident
E. Always confident

26. (If 13=A) I can comfortably breastfeed with my family members present.

A. Not at all confident
B. Not confident
C. Somewhat confident
D. Confident
E. Always confident

27. (If 13=A*)* I am satisfied with my breastfeeding experience.

A. Not at all confident

© 2025 Uscher-Pines L et al. *JAMA Network Open*.

B. Not confident
C. Somewhat confident
D. Confident
E. Always confident

28. (If 13=A) I can deal with the fact that breastfeeding can be time-consuming.

A. Not at all confident
B. Not confident
C. Somewhat confident
D. Confident
E. Always confident

29. (If 13=A) I can finish breastfeeding my baby on one breast before switching to the other breast.

A. Not at all confident
B. Not confident
C. Somewhat confident
D. Confident
E. Always confident

30. (If 13=A) I can breastfeed my baby for every feeding.

A. Not at all confident
B. Not confident
C. Somewhat confident
D. Confident
E. Always confident

31. (If 13=A) I can manage to keep up with my baby's breastfeeding demands.

A. Not at all confident
B. Not confident
C. Somewhat confident
D. Confident
E. Always confident

32. (If 13=A) I can tell when my baby is finished breastfeeding.

A. Not at all confident
B. Not confident
C. Somewhat confident
D. Confident
E. Always confident

© 2025 Uscher-Pines L et al. *JAMA Network Open*.

[Only if 9=A] The next few questions ask about challenges with breastfeeding.

33. (Only if 9=A) **In your first two weeks of breastfeeding**, did your baby have any of the following problems? Check ALL that apply.

A. My baby had trouble sucking or latching on
B. My baby choked when breastfeeding
C. My baby wouldn't wake up to breastfeed regularly enough
D. My baby was not interested in breastfeeding
E. My baby got distracted when breastfeeding
F. My baby wanted to breastfeed too often
G. My baby didn't gain enough weight or lost too much weight
H. My baby had some other problem with breastfeeding, please specify------[Programming note: Allow 300 characters]
I. My baby had no problems with breastfeeding [Programming note, if this is checked do not allow any others to be checked]

34. (Only if 9=A) **In your first two weeks of breastfeeding**, did you have any of the following problems? Check ALL that apply.

A. It took too long for my milk to come in
B. I had trouble getting the milk flow to start
C. I didn't have enough milk
D. My nipples were sore, cracked, or bleeding
E. My breasts were overfull (engorged)
F. I had a yeast infection of the breast
G. I had a clogged milk duct
H. My breasts were infected or abscessed (e.g., mastitis)
I. My breasts leaked too much
J. I had some other problem with breastfeeding, please specify-----_[Programming note: Allow 300 characters]
K. I had no problems breastfeeding [Programming note, if this is checked do not allow any others to be checked]

The next few questions ask about your experiences in the hospital or birth center where your baby was born.

35. When you were in the hospital or birth center, how often was your baby in the same room with you at night?

A. All of the time
B. Most of the time
C. Some of the time
D. None of the time
E. I don't know/don't remember

© 2025 Uscher-Pines L et al. *JAMA Network Open*.

F. I did not give birth in a hospital or birth center

36. How soon after your baby was born did you first breastfeed or pump your breast milk?
A. Within one hour
B. 1-3 hours
C. 4 or more hours
D. I don't know/don't remember
E. I did not try breastfeeding or pumping

37. When you were in the hospital or birth center, did anyone help you with breastfeeding (for example, answer your questions about breastfeeding, show you how to position your baby to breastfeed, or show you how to use a breast pump)?
A. Yes
B. No
C. I did not give birth in a hospital or birth center

38. (If 37=A) When you were in the hospital or birth center, who helped you with breastfeeding? Check ALL that apply.
A. Doctor
B. Nurse or nurse practitioner
C. Midwife
D. Lactation consultant (in-person)
E. Lactation consultant (over the phone)
F. Lactation consultant (over video)
G. Family member or friend
H. Other, please specify-------[Programming note: Allow 300 characters]

39. Since you returned home with your baby, has anyone helped you with breastfeeding?

A. Yes
B. No

40. (If 39=A) Who has helped you with breastfeeding since you returned home? Check ALL that apply.

A. Doctor
B. Nurse (for example, a home health nurse, nurse in the doctor's office)
C. Lactation consultant (in-person)
D. Lactation consultant (over the phone)
E. Lactation consultant (over video)
F. Family members or friends
G. Leader at support group or class
H. Peers at support group or class
I. WIC staff

© 2025 Uscher-Pines L et al. *JAMA Network Open*.

J. Other, please specify-------[Programming note: Allow 300 characters]

The next few questions ask about feelings that parents sometimes have after childbirth.

41. **Since your baby was born,** how often have you felt down, depressed, or sad?

A. Never
B. Rarely
C. Sometimes
D. Often
E. Always

42. **Since your baby was born,** how often have you felt restless or fidgety?

A. Never
B. Rarely
C. Sometimes
D. Often
E. Always

43. **Since your baby was born,** how often have you felt panicky?

A. Never
B. Rarely
C. Sometimes
D. Often
E. Always

The next few questions ask about COVID-19 vaccines.

44. Have you received one or more doses of COVID-19 vaccine?

A. Yes
B. No

45. [If 44=A] When did you receive your first dose of the COVID-19 vaccine?

A. Before I became pregnant
B. During pregnancy
C. After my baby was born

46. [If 44=A] During what month and year did you receive your first dose of COVID-19 vaccine? Please give your best guess. Month ________ Year ________

47. [If 44=A] What types of COVID-19 vaccines have you received? Check ALL that apply.

A. One dose of Pfizer vaccine
B. Two or more doses of Pfizer vaccine

© 2025 Uscher-Pines L et al. *JAMA Network Open*.

C. One dose of Moderna vaccine
D. Two or more doses of Moderna vaccine
E. Johnson and Johnson (J&J) vaccine
F. Other
F. I don't know

48. [If 44=B] There are different reasons why people do not get vaccinated for COVID-19. What are the main reasons why you have not received a COVID-19 vaccine? Please check up to three reasons.

A. I don't need it
B. I already had COVID-19
C. I may experience side effects or become sick
D. I am worried it could harm my baby
E. I am worried it could affect my fertility
F. The vaccine was not tested enough in pregnant women
G. I dislike needles
H. I didn't get around to it / I did not have the time
I. I don't think that COVID-19 vaccines work
J. I had trouble getting an appointment or travelling to an appointment
K. Other, please specify ________

49. [If 44=B] Do you think you will receive a COVID-19 vaccine in the future?

A. I will definitely not get vaccinated
B. I will only get vaccinated if it is required
C. I want to wait and see
D. I want to wait until I am done breastfeeding
E. I plan to get vaccinated within the next three months

Please rate how much you agree with the next two statements.

50. Getting vaccinated for COVID-19 <u>during pregnancy</u> is safe for me and my baby.

A. Strongly Disagree
B. Disagree
C. Neither agree nor disagree
D. Agree
E. Strongly Agree

51. Getting vaccinated for COVID-19 <u>while breastfeeding</u> is safe for me and my baby.

A. Strongly Disagree
B. Disagree
C. Neither agree nor disagree
D. Agree
E. Strongly Agree

© 2025 Uscher-Pines L et al. *JAMA Network Open*.

52. [If 45=B or C] What impact did pregnancy have on your decisions about COVID-19 vaccination? Check ALL that apply.

A. Being pregnant made me more interested in getting vaccinated
B. Being pregnant encouraged me to get vaccinated earlier than I would have otherwise
C. Being pregnant made me less interested in getting vaccinated
D. Being pregnant made me delay getting vaccinated for weeks or months
E. Pregnancy had no impact on my decisions about vaccination

(Intervention arm only)

The next few questions ask about your experience with the free video calls with lactation consultants using the PACIFY app. Please answer these questions even if you have not used the service.

47) Have you participated in any video calls with a lactation consultant using the PACIFY app?

A. Yes
B. I tried to but didn't complete a video call
C. No

48) (If 47=C) Why haven't you used the PACIFY app for a video call? Check ALL that apply.

A. I haven't had any breastfeeding problems
B. I stopped breastfeeding
C. I lost my phone or my phone stopped working
D. I couldn't download the app
E. I forgot about the free video calls
F. I don't think the service will help me
G. I am too busy
H. I am not sure what to use the service for
I. I am not comfortable using the service
J. I could not find access to wireless internet or cellular service
K. I could not find a private place to do the video call
L. Other, please explain-------_____[Programming note: Allow 300 characters]

© 2025 Uscher-Pines L et al. *JAMA Network Open*.

49) (If 47=C) Do you think you will use the PACIFY app in the next several months (before your baby is 6 months old)?

A. Yes
B. No

50) (If 47=C AND 549=B AND 48 does not equal B) Why don't you think you will use it? Please explain __________________ [Programming note: Allow 300 characters]

51) (If 47=B) Did you have any technical problems using the app (e.g., poor connection)? Please describe_________________[Programming note: Allow 300 characters]

52) (If 47=B) What questions were you hoping to discuss with the lactation consultant through the app? Please describe________________[Programming note: Allow 300 characters]

53) (If 47=B) Do you plan to try to use the PACIFY app in the next several months?

A. Yes
B. No

54) (If 47=B and 53=B) Why don't you think you will use it? Please explain_______________[Programming note: Allow 300 characters]

55) (If 47=A) How many times have you used the PACIFY app to do video calls with lactation consultants?

A. 1
B. 2
C. 3
D. 4 or more times

56) (If 47=A) What did you discuss with the lactation consultant on your video call(s)? Please list them___________________________[Programming note: Allow 500 characters]

57) (If 47=A) Did you have any of the following problems or challenges during your video call(s)? Check ALL that apply.

A. I had no problems or challenges
B. Trouble finding wireless internet or cellular network
C. Trouble connecting to wireless internet or cellular network

© 2025 Uscher-Pines L et al. *JAMA Network Open*.

D. Trouble finding a private place to do the visit
E. Difficulty positioning phone or other device
F. Trouble with the connection once the call started (e.g., dropped calls)
G. Long wait time for lactation consultant
H. Other, please specify________[Programming note: Allow 300 characters]

58) (If 47=A) Thinking about all of the video calls you have had over the past few weeks, how helpful was the breastfeeding help you received through the PACIFY app?

A. Not at all helpful
B. Somewhat helpful
C. Neither helpful nor unhelpful
D. Helpful
E. Very helpful

59) (If 47=A) Would you recommend the PACIFY app to a friend or relative with similar issues or questions?

A. Yes
B. No

60) (If 47=A) Do you have any recommendations for improving the PACIFY app? If so, please describe ________[Programming note: Allow 500 characters]

Thank you for completing the survey! Your $20 gift code will be sent to you shortly.

© 2025 Uscher-Pines L et al. *JAMA Network Open*.

Week 24 Survey

Introduction

This is the last survey that you will complete as part of the Tele-MILC study. This survey asks about your experience feeding your baby over the last six months. After you complete the survey, you will receive a $20 gift card through your email.

Please answer all questions honestly. There is no wrong way to answer these questions, and no answer will disqualify you from receiving your gift card.

----

1. Is your baby living with you now? (If you have been separated from your baby permanently or for many weeks due to work related travel, adoption, death, or any other reason please check "No.")

   A. Yes
   B. No [If not, terminate and provide incentive]

[IF Q1=B] This is the last question. Thank you for taking the time to complete this survey. Your $20 gift code will be sent to you shortly.

2. Was your baby ever breastfed or fed your breast milk with a bottle, even for a short period of time?

   A. Yes
   B. No

3. (Only if 2=A) In the past week, was your baby breastfed or fed your breast milk with a bottle?

   A. Yes
   B. No

----

4. (If 3=A) How are you providing breast milk to your baby?
   A. Only feeding from the breast
   B. Only giving pumped breast milk
   C. A combination of feeding from the breast and giving pumped breast milk

5. (If 3=A) How old was your baby when he/she was first fed formula? Please give your best guess.

   A. My baby has not been fed formula
   B. < 7 days
   C. 8-30 days
   D. 1-3 months

© 2025 Uscher-Pines L et al. *JAMA Network Open*.

E. 4-5 months
F. 6 months or more

6. (If 3=A) In the last 24 hours, how much formula was your baby fed? Please give your best guess.

A. None: my baby was not fed any formula in the last 24 hours
B. Less than 7 ounces
C. 7-12 ounces
D. 13-22 ounces
E. 23 or more ounces

7. In the last 24 hours, what proportion of your baby's total diet were the following items? Please give your best guess. [Programming note- should add up to 100%. Add prompt if it does not add up- Note that if does not, they should not be forced to correct it.)

A. Breast milk	%
C. Formula	%
D. Baby food (e.g., cereal, pureed vegetables)	%
E. Other, please specify--- _____	%
	100%

8. (If 3=A) **Since your baby was born**, did <u>your baby</u> have any of the following problems with breastfeeding**?** Check ALL that apply.

A. My baby had trouble sucking or latching on
B. My baby choked while breastfeeding
C. My baby wouldn't wake up to breastfeed regularly enough
D. My baby was not interested in breastfeeding
E. My baby got distracted when breastfeeding
F. My baby wanted to breastfeed too often
G. My baby didn't gain enough weight or lost too much weight
H. My baby had some other problem, please specify_[Programming note: Allow 300 characters]
I. My baby had <u>no</u> problems with breastfeeding [Programming note: If this is selected, do not allow other selections]

9**.** (If 3=A) **Since your baby was born**, did <u>you</u> have any of the following problems with breastfeeding**?** Check ALL that apply.

© 2025 Uscher-Pines L et al. *JAMA Network Open*.

A. It took too long for my milk to come in
B. I had trouble getting the milk flow to start
C. I didn't have enough milk
D. My nipples were sore, cracked, or bleeding
E. My breasts were overfull (engorged)
F. I had a yeast infection of the breast
G. I had a clogged milk duct
H. My breasts were infected or abscessed
I. My breasts leaked too much
J. I had some other problem, please specify_[Programming note: Allow 300 characters]
J. I had <u>no</u> problems breastfeeding [Programming note: If this is selected, do not allow other selections]

(If 3=A) The next few questions ask you to describe your overall experience breastfeeding since your baby was born. Please rate how much you agree with the statements below.

10. (If 3=A) In general, I believe my baby is satisfied with breastfeeding.
    F. Strongly Disagree
    G. Disagree
    H. Neither agree nor disagree
    I. Agree
    J. Strongly Agree

11. (If 3=A) In general, I am satisfied with breastfeeding.
    A. Strongly Disagree
    B. Disagree
    C. Neither agree nor disagree
    D. Agree
    E. Strongly Agree

12. (If 3=A) I become more relaxed as I sit and breastfeed.
    A. Strongly Disagree
    B. Disagree
    C. Neither agree nor disagree
    D. Agree
    E. Strongly Agree

13. (If 3=A) My baby appears to enjoy breastfeeding.
    A. Strongly Disagree
    B. Disagree
    C. Neither agree nor disagree
    D. Agree
    E. Strongly Agree

14. (If 3=A) In general, I feel successful at breastfeeding my baby.
    A. Strongly Disagree
    B. Disagree

© 2025 Uscher-Pines L et al. *JAMA Network Open*.

C. Neither agree nor disagree
D. Agree
E. Strongly Agree

----

15. (If 3=B) How old was your baby when you completely stopped feeding him/her your breast milk? Please give your best guess.

A. I never fed my baby my breastmilk
B. Less than 1 month
C. 1 month
D. 2 months
E. 3 months
F. 4 months
G. 5 months
H. 6 months or more

16. (If 3=B) What were your reasons for stopping breastfeeding? Check your top three reasons.

A. My baby had difficulty sucking or latching
B. I thought my baby was not gaining enough weight
C. Breastfeeding was painful
D. Breastfeeding was too hard or too time consuming
E. Breast milk alone did not satisfy my baby
F. I was not making enough milk or my milk dried up
G. I had too many other duties or responsibilities
H. I felt it was the right time to stop breastfeeding
I. I got sick or I had to stop for medical reasons
J. I needed to change my diet
K. I needed to take a medication that interfered with breastfeeding
L. I started smoking or drinking alcohol
M. My baby lost interest in breastfeeding
N. I had to go back to work or school
O. My baby got sick or had a medical issue
P. Family and/or friends told me I should stop breastfeeding
Q. A doctor or other medical professional told me I should stop breastfeeding
R. Other, please specify____________________[Programming note: Allow 300 characters]

17. (If 3=B) **During the time you were breastfeeding your baby,** did your baby have any of the following problems? Check ALL that apply.

A. My baby had trouble sucking or latching on
B. My baby choked while breastfeeding

© 2025 Uscher-Pines L et al. *JAMA Network Open*.

C. My baby wouldn't wake up to breastfeed regularly enough
D. My baby was not interested in breastfeeding
E. My baby got distracted when breastfeeding
F. My baby breastfed too often
G. My baby didn't gain enough weight or lost too much weight
H. My baby had some other problem, please specify__[Programming note: Allow 300 characters]
I. My baby had <u>no</u> problems with breastfeeding [Programming note, if this is checked do not allow any others to be checked]

18. (If 3=B) **During the time you were breastfeeding your baby,** did <u>you</u> have any of the following problems? Check ALL that apply.

A. It took too long for my milk to come in
B. I had trouble getting the milk flow to start
C. I didn't have enough milk
D. My nipples were sore, cracked, or bleeding
E. My breasts were overfull (engorged)
F. I had a yeast infection of the breast
G. I had a clogged milk duct
H. My breasts were infected or abscessed
I. My breasts leaked too much
K. I had some other problem, please specify_[Programming note: Allow 300 characters]
L. I had <u>no</u> problems with breastfeeding [Programming note, if this is checked do not allow any others to be checked]

(If 3=B) The next few questions ask you to think back to when you were breastfeeding and consider how you felt at the time. Please rate how much you agree with the statements below.

19. (If 3=B) In general, I believed my baby was satisfied with breastfeeding.
   A. Strongly Disagree
   B. Disagree
   C. Neither agree nor disagree
   D. Agree
   E. Strongly Agree

20. (If 3=B) In general, I was satisfied with breastfeeding.
   A. Strongly Disagree
   B. Disagree
   C. Neither agree nor disagree
   D. Agree
   E. Strongly Agree

21. (If 3=B) I became more relaxed as I sat and breastfed.
   A. Strongly Disagree

© 2025 Uscher-Pines L et al. *JAMA Network Open*.

B. Disagree
C. Neither agree nor disagree
D. Agree
E. Strongly Agree

22. (If 3=B) My baby appeared to enjoy breastfeeding.
A. Strongly Disagree
B. Disagree
C. Neither agree nor disagree
D. Agree
E. Strongly Agree

23. (If 3=B) In general, I felt successful at breastfeeding my baby.
A. Strongly Disagree
B. Disagree
C. Neither agree nor disagree
D. Agree
E. Strongly Agree

24. Has your baby ever been fed another person's breast milk (e.g., donor milk, shared milk)?

A. Yes
B. No

25. (If 24=A) Not including your own breast milk, what types of breast milk has your baby been fed? Check ALL that apply.

A. Donor breast milk from a milk bank
B. Breast milk from a milk sharing network (e.g., Human Milk 4 Human Babies)
C. Breast milk from a friend or family member
D. Other, please describe [Programming note: Allow 300 characters]
E. I don't know

26. How likely is it that you would breastfeed if you had another child?

A. Very unlikely
B. Unlikely
C. Neutral
D. Likely
E. Very likely

(If 2=A) The next few questions ask about the breastfeeding help you have had since returning home from the hospital or birth center where you delivered your baby. If you did not give birth in a hospital or birth center, answer these questions thinking back on the time since you gave birth.

© 2025 Uscher-Pines L et al. *JAMA Network Open*.

27. (If 2=A) Since you returned home, has anyone helped you with breastfeeding (for example, answered your questions about breastfeeding, showed you how to position your baby to breastfeed, or showed you how to use a breast pump)?

A. Yes
B. No

28. (If 27=A) Who has helped you with breastfeeding since you returned home? Check ALL that apply.

A. Doctor
B. Nurse (for example, a home health nurse, nurse at doctor's office)
C. Lactation consultant (in-person)
D. Lactation consultant (over the phone)
E. Lactation consultant (over video)
F. Family members or friends
G. Leader at support group or class
H. Peers at support group or class
I. WIC staff
J. Other, please specify____________________[Programming note: Allow 300 characters]

The next few questions ask about your opinions of healthcare providers. Please rate how much you agree with the statements below. Healthcare providers include doctors, nurses, and other providers who see patients. Some of these questions are sensitive. We are asking them because discrimination can negatively impact your health.

29. Sometimes health care providers care more about what is convenient for them than about their patients' medical needs.

A. Strongly agree
B. Agree
C. Neither agree nor disagree
D. Disagree
E. Strongly disagree

30. Health care providers are extremely thorough and careful.

A. Strongly agree
B. Agree
C. Neither agree nor disagree
D. Disagree
E. Strongly disagree

© 2025 Uscher-Pines L et al. *JAMA Network Open*.

31. I completely trust health care providers' decisions about which medical treatments are best.

A. Strongly agree
B. Agree
C. Neither agree nor disagree
D. Disagree
E. Strongly disagree

32. A health care provider would never mislead me about anything.

A. Strongly agree
B. Agree
C. Neither agree nor disagree
D. Disagree
E. Strongly disagree

33. Overall, I trust health care providers completely.

A. Strongly agree
B. Agree
C. Neither agree nor disagree
D. Disagree
E. Strongly disagree

34. Healthcare providers pay less attention to me because of my race or ethnicity.

A. Strongly agree
B. Agree
C. Neither agree nor disagree
D. Disagree
E. Strongly disagree

35. Healthcare providers discriminate against me because of my race or ethnicity.

A. Strongly agree
B. Agree
C. Neither agree nor disagree
D. Disagree
E. Strongly disagree

The next few questions ask about your work status.

36. Are you currently working for pay?

© 2025 Uscher-Pines L et al. *JAMA Network Open*.

A. Yes
B. No

37. (If 36=A) How many hours do you work per week?

A. 40 hours or more
B. 30-39 hours
C. 20-29 hours
D. 10-19 hours
E. Under 10 hours

38. (If 36=A) How old was your baby when you began working for pay? Please give your best guess.

A. Less than 1 week
B. 1-4 weeks
C. 5-8 weeks
D. 9-12 weeks
E. 13 or more weeks

The next few questions ask about the people in your life including your family, friends, and neighbors.

39. How many people can you count on if you have personal problems? Would you say….

A. None
B. 1 to 2
C. 3 to 5
D. 5 or more

40. How easy is it to get practical help (for example, a ride home from the doctor, help moving a couch) from people in your life if you need it?

A. Very difficult
B. Difficult
C. Possible
D. Easy
E. Very easy

[Intervention Only]

The next few questions ask you about your experience with the free video calls with lactation consultants using the PACIFY app. Please answer these questions even if you have not used the service.

© 2025 Uscher-Pines L et al. *JAMA Network Open*.

41. Since the study began, have you seen any breastfeeding videos from PACIFY on social media (Facebook or Instagram)?

A. Yes
B. No

42. [If 41=A] Thinking about all of the breastfeeding videos from PACIFY that you saw on social media, how helpful was the advice they provided?

A. Not at all helpful
B. Somewhat helpful
C. Neither helpful nor unhelpful
D. Helpful
E. Very helpful

43**)** Since the study began, how many times did you participate in video calls using the PACIFY app?

A. None: I didn't have any video calls with lactation consultants
B. 1
C. 2
D. 3
E. 4 or more

44) (If 43=A) Why didn't you use the PACIFY app to do video calls? Check ALL that apply.

A. I stopped breastfeeding
B. I haven't had any breastfeeding problems
C. I lost my phone or my phone stopped working
D. I lost my code to sign up
E. I forgot about the free video calls
F. I didn't think the video calls would help me
G. I was too busy
H. I was not sure what to use the video calls for
I. I was not comfortable using the video calls
J. I could not find access to wireless internet or cellular service
K. I could not find a private place to do a video call
L. I could not get the app to work
M. Other, please explain-------____[Programming note: Allow 300 characters]

© 2025 Uscher-Pines L et al. *JAMA Network Open*.

45) (If 43=B) How old was your baby when you used the PACIFY app? Please give your best guess.

_____Weeks

46) (If 43=B) Why didn't you use the PACIFY app again? Check ALL that apply.

A. I stopped breastfeeding
B. I didn't have any additional breastfeeding problems or questions
C. I lost my phone or my phone stopped working
D. I forgot about the free video calls
E. I didn't think another video call would help me
F. I had a bad experience with my first video call
G. I was too busy
H. It was too much trouble to use the video call
I. Other, please explain____[Programming note: Allow 300 characters]

47) (If 43=C, D, or E) How old was your baby when you used the PACIFY app for the first time? Please give your best guess.

_____Weeks

48) (If 43= B, C, D, or E) What did you discuss with the lactation consultant using the PACIFY app? Please describe_________________[Programming note: Allow 500 characters]

49) (If 43= B, C, D, or E) Thinking about all of the video calls you have had, how helpful was the breastfeeding help you received through the PACIFY app?

A. Not at all helpful
B. Somewhat helpful
C. Neither helpful nor unhelpful
D. Helpful
E. Very helpful

50) (If 43= B, C, D, or E) Thinking about all the video calls you have had, how satisfied were you with the breastfeeding help you received through the PACIFY app?

A. Not at all satisfied
B. Somewhat satisfied
C. Neither satisfied nor unsatisfied
D. Satisfied

© 2025 Uscher-Pines L et al. *JAMA Network Open*.

E. Very satisfied

51) (If 43= B, C, D, or E) Would you recommend the PACIFY app to a friend or relative with breastfeeding questions or problems?

A. Yes
B. No

52) (If 43= B, C, D, or E) Do you have any recommendations for improving the PACIFY app? Please describe ___________________ [Programming note: Allow 500 characters]

Thank you for completing the survey! Your $20 gift code will be sent to you shortly.

© 2025 Uscher-Pines L et al. *JAMA Network Open*.
