# Supplementary material for: Telelactation Services and Breastfeeding by Race and Ethnicity: A Randomized Clinical Trial
Source: JAMA Netw Open. 2025 Feb 27;8(2):e2461958. doi: 10.1001/jamanetworkopen.2024.61958 (PMC11868977; doi:10.1001/jamanetworkopen.2024.61958)
Supplement: Supplement 3. — Data Sharing Statement [file jamanetwopen-e2461958-s003.pdf]

Data Sharing Statement

Uscher-Pines. Telelactation Services and Breastfeeding by Race and Ethnicity. *JAMA Netw Open*. Published February 27, 2025. doi:10.1001/jamanetworkopen.2024.61958

Data
Additional Information: ClinicalTrials.gov Identifier: NCT04856163
Data available: Yes
Data types: Deidentified participant data, Data dictionary
How to access data: Available from lead author upon request. Email luscherp@rand.org
When available: With publication

Supporting Documents
Document types: None

Additional Information
Who can access the data: Researchers whose proposed use has been approved.
Types of analyses: For research purposes.
Mechanisms of data availability: After approval from PI and with IRB approval.
